# Genetic regulation of injury-induced heterotopic ossification in adult zebrafish

**DOI:** 10.1242/dmm.050724

**Published:** 2024-05-31

**Authors:** Arun-Kumar Kaliya-Perumal, Cenk Celik, Tom J. Carney, Matthew P. Harris, Philip W. Ingham

**Affiliations:** ^1^ Lee Kong Chian School of Medicine, Nanyang Technological University, 59 Nanyang Drive 636921, Singapore; ^2^ Department of Genetics, Evolution and Environment, Genetics Institute, University College London, London WC1E 6BT, UK; ^3^ Institute of Molecular and Cell Biology (IMCB), Agency for Science, Technology and Research (A*STAR), 61 Biopolis Drive, Proteos 138673, Singapore; ^4^ Department of Genetics, Harvard Medical School, Boston, MA 02115, USA; ^5^ Department of Orthopedic Research, Boston Children's Hospital, Boston, MA 02115, USA; ^6^ Department of Life Sciences, University of Bath, Bath BA2 7AY, UK

**Keywords:** Contusion, Heterotopic ossification, Interleukin 11, Myositis ossificans, Potassium channels

## Abstract

Heterotopic ossification is the inappropriate formation of bone in soft tissues of the body. It can manifest spontaneously in rare genetic conditions or as a response to injury, known as acquired heterotopic ossification. There are several experimental models for studying acquired heterotopic ossification from different sources of damage. However, their tenuous mechanistic relevance to the human condition, invasive and laborious nature and/or lack of amenability to chemical and genetic screens, limit their utility. To address these limitations, we developed a simple zebrafish injury model that manifests heterotopic ossification with high penetrance in response to clinically emulating injuries, as observed in human myositis ossificans traumatica. Using this model, we defined the transcriptional response to trauma, identifying differentially regulated genes. Mutant analyses revealed that an increase in the activity of the potassium channel Kcnk5b potentiates injury response, whereas loss of function of the interleukin 11 receptor paralogue (Il11ra) resulted in a drastically reduced ossification response. Based on these findings, we postulate that enhanced ionic signalling, specifically through Kcnk5b, regulates the intensity of the skeletogenic injury response, which, in part, requires immune response regulated by Il11ra.

## Teaser

**Editor's choice:** A zebrafish injury model demonstrates heterotopic bone formation resembling human myositis ossificans traumatica, suggesting potential osteo-induction and ectopic bone growth post mechanistically relevant injuries.

## INTRODUCTION

The musculoskeletal system is an intricate association of muscles, tendons, ligaments, bones and joints that together support posture and enable locomotion and protection (Roberts, 2002). The state of this construction is far from static; rather, through integration of strain, localised new bone can be formed to accommodate differential strain, turnover or repair (Bergmann et al., 2010; Watanabe-Takano et al., 2021). When these remodelling signals are aberrant or enhanced, ectopic growth of bone can result. If the enhanced bony growth involves the joints and the associated structural connectivity of the skeleton, this can lead to severe effects on quality of life for patients. The disorders causing ectopic bone growth can broadly be classified into genetic and acquired types. The genetic types harbour an underlying mutation driving the mechanism of extensive heterotopic ossification, whereas acquired types are purely injury induced and limited to the site of injury (Meyers et al., 2019).

Myositis ossificans traumatica (MOT), the most common form of acquired heterotopic ossification observed primarily in young adults during the second or third decade of life, typically follows a benign course and is triggered by a single traumatic incident or repeated injuries to the same anatomical site. Various musculoskeletal traumas such as fractures, muscle contusions, wartime wounds, burns and surgeries have shown development of heterotopic ossification at the site of injury (Ahrengart, 1991; Andreu Martinez et al., 2007; Beiner and Jokl, 2002; Forsberg et al., 2009). The quadriceps femoris muscle and the brachialis muscle are the most frequently reported sites (Saad et al., 2021). In addition, repeated injuries, for example, those seen in horseback riders, have been shown to lead to the development of ectopic bone, referred to as ‘rider's bone’, in the origin of the adductors of the thigh (Binnie, 1903; Rosenstirn, 1918). Similarly, shooters (e.g. people who use rifles) may develop ectopic bone in the deltoid muscle, known as ‘shooter's bone’ (Walczak et al., 2015). Neurogenic heterotopic ossification, in contrast, involves the occurrence of heterotopic ossification around a fracture or a contusion site, especially around the hip joint, whenever there is a concomitant traumatic brain or spinal cord injury (Genet et al., 2009; Sakellariou et al., 2012).

These injury-associated conditions are quite instructive as the underlying mechanism of ectopic bone formation is believed to follow a particular trend. Firstly, following a muscle or bony injury, there is infiltrative bleeding and inflammation (Beiner and Jokl, 2002). The subsequent cascade of events normally results in either complete anatomical healing or scar formation. However, the dysregulation of signalling of the cells at the site of injury can lead to their inappropriate differentiation into chondrocytes or osteoblasts, ultimately resulting in heterotopic bone (Kan et al., 2009). Once triggered, little can be done to alter the natural course of the disorder. Worse still, invasive procedures can enhance ongoing inflammation and lead to excessive heterotopic bone formation. Therefore, management is typically conservative and excision is only recommended after the heterotopic bone fully matures (Meyers et al., 2019). By that time, however, stiffness and muscle contractures have already developed, limiting full recovery.

In contrast to acquired heterotopic ossification, genetic variants show extreme systemic heterotopic ossification. Fibrodysplasia ossificans progressiva (FOP), for instance, is a heritable disorder characterised by heterotopic ossification predominantly at the muscles, tendons, ligaments and fascia (Kaplan et al., 2008). Similarly, progressive osseous hyperplasia and Albright's hereditary osteodystrophy are characterised by heterotopic ossification predominantly at cutaneous and subcutaneous sites (Kaplan and Shore, 2000). FOP has been the subject of comprehensive research and has yielded significant insights into the formation of heterotopic bone, highlighting the role of local signals (Pignolo et al., 2013). In patients with FOP, a gain-of-function mutation in the *ACVR1* gene, encoding the activin A receptor type 1, a bone morphogenetic protein (BMP) type 1 receptor, drives the heterotopic ossification process by aberrantly initiating both ligand-dependent and -independent activation of the BMP signalling cascade (Hino et al., 2015; Kaplan et al., 2012; Pang et al., 2016), a response coincident with local tissue damage and inflammation (Matsuo et al., 2019).

As the underlying mechanisms of both genetic and acquired heterotopic ossification are closely related, identification of common targets for intervention is plausible, allowing alleviation of growth without inducing added insult or inflammation. Although current research on FOP has led to the discovery of certain drugs that are undergoing clinical trials (Pignolo et al., 2022, 2023), it remains unlikely that these would be effective for damage-induced exposures, given the abnormal enhanced sensitivity seen in the gain-of-function condition of FOP etiology. In light of the higher incidence of acquired heterotopic ossification occurring in standard injuries, it is important to address the etiology of these disorders and bridge our knowledge gap concerning the mechanisms underlying the tissue response specific to this injury-induced pathology. This requires the utilisation of animal models of injury-induced heterotopic ossification, of which there are a few examples (Anthonissen et al., 2014; Cappato et al., 2020; Dey et al., 2017; Kan and Kessler, 2011). These include a rabbit model of immobilised knee manipulation (Bartlett et al., 2006; Michelsson et al., 1994), an Achilles tenotomy model in rats and mice (Buck, 1953; McClure, 1983), simulation of hip arthroplasty by surgical reaming of rabbit femurs (Schneider et al., 1998), injection of irritant substances such as 40% ethanol or acid alcohol into rabbit muscles (Heinen et al., 1949; Selle and Urist, 1961), direct implantation of BMP2- or BMP4-containing matrix in mice (Glaser et al., 2003; Lounev et al., 2009), stripping the periosteum and damaging the overlying muscles of the femur in dogs (Collins et al., 1965), and crush injury to the muscles overlying the femur in sheep (Walton and Rothwell, 1983). Recently, a combination of hindlimb amputation and cardiotoxin injection was shown to induce heterotopic ossification in mice (Cao et al., 2023). The most intricate of all is a sheep model simulating battlefield trauma, featuring high-power blast exposure and subsequent bone damage, followed by tourniquet application, bacterial exposure and negative pressure wound therapy, resulting in heterotopic ossification (Epperson et al., 2021). Although some of these models replicate specific modes of heterotopic ossification occurrence in humans, others lack mechanistic relevance or their procedures are invasive and laborious, making them unsuitable for broad experimental analysis. An established and broadly applicable injury-induced heterotopic ossification model is thus still lacking.

Zebrafish are amenable to experimental manipulation, such as surgical extirpation as well as generation of damage models in the study of repair and regeneration, and as such present an attractive laboratory organism for the development of an experimental platform for heterotopic ossification. Notably, the powerful genetics in zebrafish facilitate investigation of the genetic and cellular activity underlying these responses. There are remarkable parallels between zebrafish and mammals in their musculoskeletal development and remodelling. In particular, zebrafish skeletal cells, the patterns of ossification and the sequential transcriptional hierarchy driving osteogenesis share striking similarities with those observed in mammals (Kaliya-Perumal and Ingham, 2022). In the last decade, the zebrafish has become an important organism for the study of skeletogenesis, particularly in mature tissues, serving as models of skeletal dysplasia such as osteogenesis imperfecta, osteopetrosis and a broad range of structural congenital disorders (Mari-Beffa et al., 2021). The zebrafish has also been used to model the genetic signalling in FOP (Allen et al., 2020; LaBonty et al., 2017, 2018).

Here, we investigated the efficiency and characteristics of experimental induction of heterotopic ossification in the adult zebrafish, specifically to emulate mechanistically relevant injuries causing these disorders in humans. We used this model to assess the genetic regulation of this response and novel targets for its intervention.

## RESULTS

### Exploring heterotopic ossification in the caudal peduncle and tail fin

**Fig. 1.**
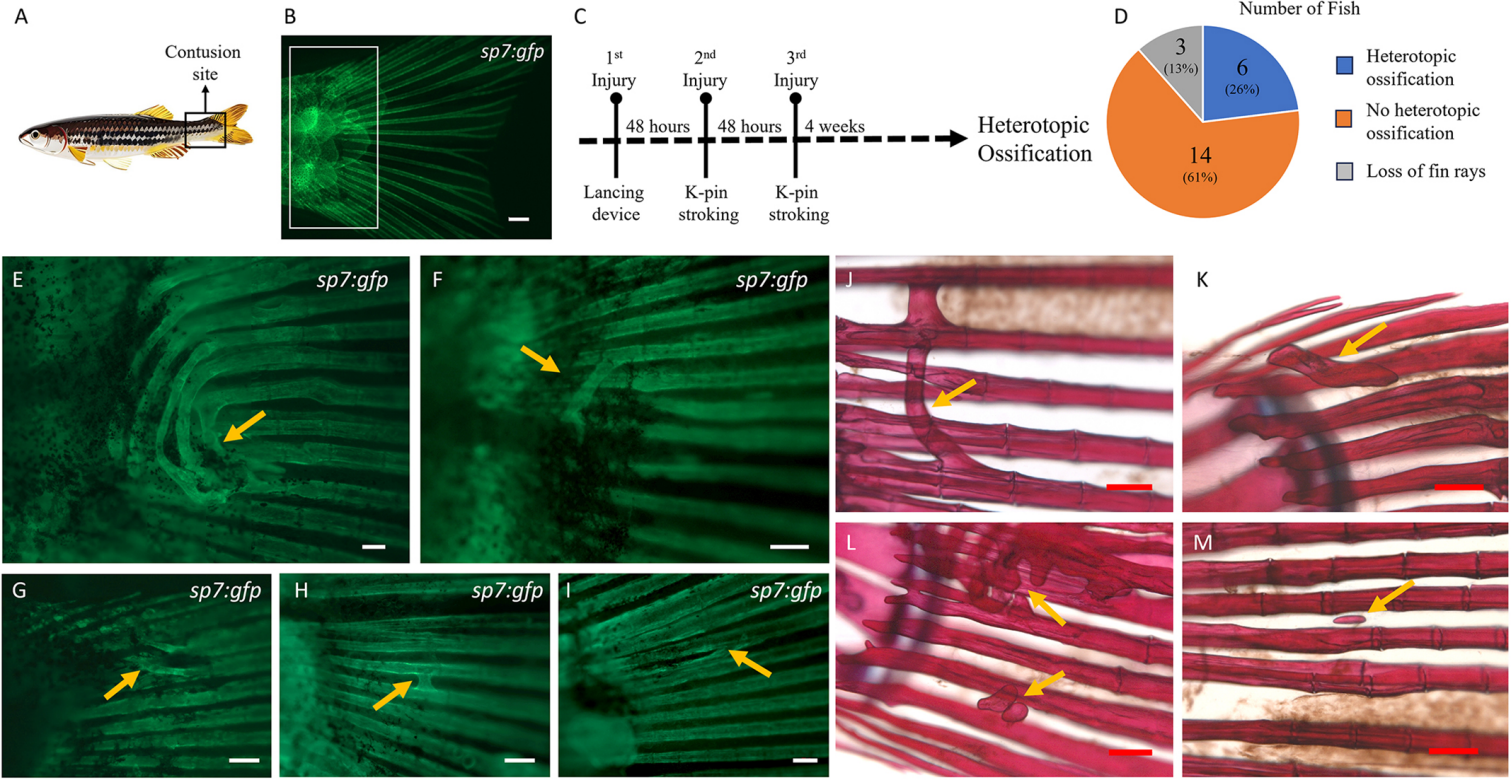
**Heterotopic bone formation at the tail fin lepidotrichia.** (A) Site of caudal peduncle contusion (boxed area). (B) Appearance of an uninjured tail fin under fluorescence microscopy in a transgenic *Tg(sp7:gfp)* zebrafish. The boxed area represents the site where heterotopic bone formation was noted, as shown in E-M. (C) Timeline representing the recurring injuries with a 48-h interval in between, followed by a 4-week wait to observe heterotopic bone. (D) Pie chart showing the overall number of fish with and without heterotopic bone (*n*=23). (E-I) Fluorescence microscopy of injured *Tg(sp7:gfp)* zebrafish. (J-M) Alizarin Red staining of injured wild-type zebrafish. Exuberant heterotopic bone formation (arrow in E) bridging multiple adjacent fin rays beneath the overlying soft tissues of the caudal peduncle and various other forms of heterotopic bone (arrows in F-M) were observed. Scale bars: 500 µm (B,E); 200 µm (F-M).

The zebrafish has become an established model system for the analysis of human disease genetics and, within the last decade, has been increasingly exploited as a model for diseases that affect adult structures, including the skeleton (Carnovali et al., 2019; Valenti et al., 2020). We speculated that by employing relevant injury methods, heterotopic ossification could manifest in zebrafish adults. To pursue this, we initially chose the caudal peduncle region just rostral to the tail fin as a site wherein mechanistically relevant contusion or fracture-contusion injuries could be inflicted, mirroring those occurring in humans (Fig. 1A). This region of the fish is muscular, remote from essential organ functions, and adjacent to articulation of the tail fin bones with the hypurals and parahypurals. In addition, this area favours live fluorescence imaging of the skeleton utilising transgenic reporters such as *Tg(sp7:gfp*), which drives GFP expression in early osteoblasts throughout the body. Hence, the entire skeleton exhibits bright green fluorescence, including heterotopic ossification sites starting from early stages. All experimental fish were imaged prior to injuries to rule out any anatomical variations. Images revealed the tail fin lepidotrichia to be clearly visible, extending to the proximal region beneath the overlying soft tissue of the caudal peduncle (Fig. 1B). However, the hypural, parahypural and the connection points of these bones with the fin rays remained obscured by the surrounding soft tissue cover and were not visualised.

A lancing device was designed for the controlled induction of initial contusions (Fig. S1). Observing resolution of contusion within 48 h in all the fish, the injury site was gently stroked with a blunt-tip Kirschner pin (K-pin) to revert it to its contused state. This process was repeated twice after the first injury, sustaining the contusion phase for up to 1 week (Fig. 1C). After a 4-week interval, live imaging was performed and observations were noted (Fig. 1D). New bone formation resembling heterotopic ossification was observed at the caudal margin of the contusion site (Fig. 1E-M), predominantly originating from the lepidotrichia beneath the contused muscle and occupying the adjacent soft tissue area. Although this site fell outside the trajectory of the lancet strikes, the resulting contusions spread across a wider area, including the region where ectopic bone was noted. In one of these fish, robust bridging bone formation was noted, connecting multiple adjacent fin rays (Fig. 1E). However, the overall occurrence rate was found to be minimal (26.1%, *n*=23) (Fig. 1D). Hence, it became imperative to identify a more suitable and pertinent location with higher penetrance for further research.

### Intermuscular bone hypertrophy at contusion sites

**Fig. 2.**
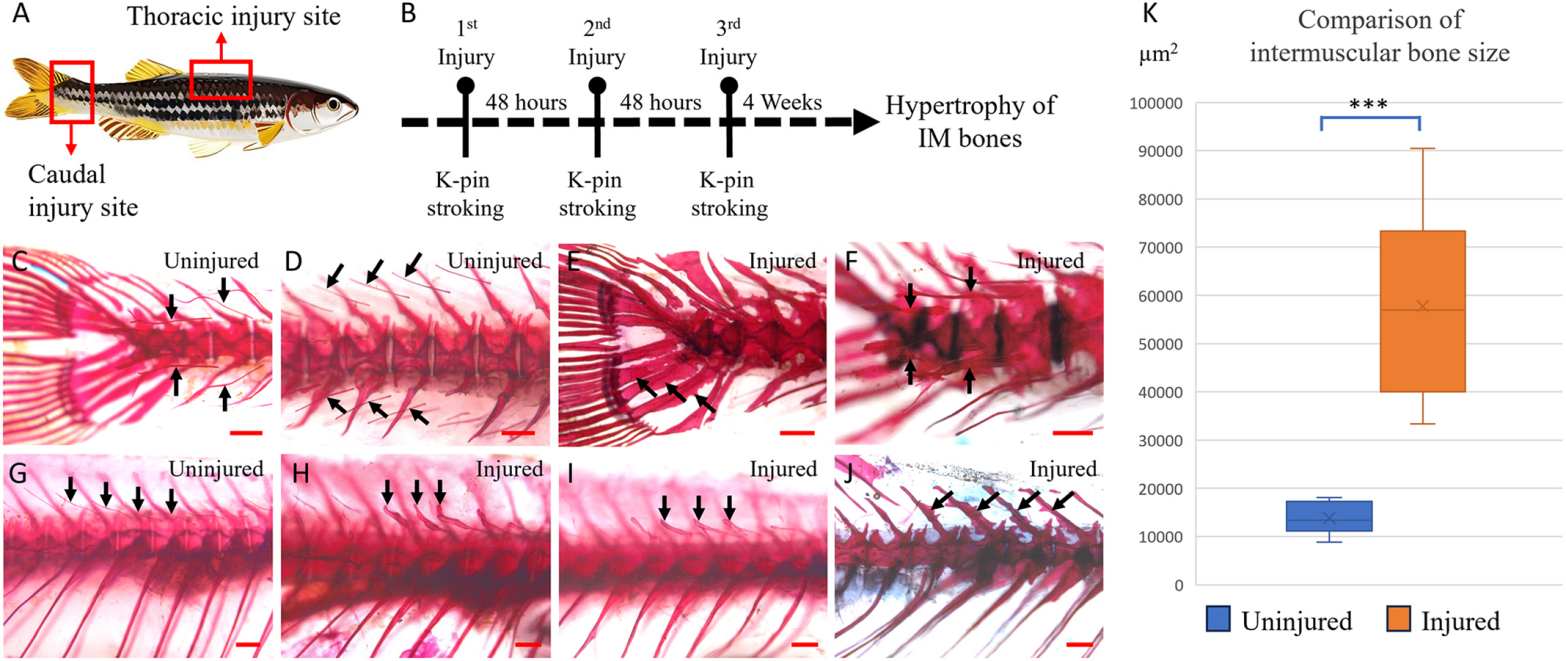
**Hypertrophy of intermuscular bones following contusions.** (A) Boxed areas represent the contusion sites. (B) Injury timeline, similar to that for pectoral fin injuries but resulting in hypertrophy of intermuscular (IM) bones. (C-F) Alizarin Red-stained caudal peduncle regions of wild-type fish. (G-J) Alizarin Red-stained thoracic regions of wild-type fish. Scale bars: 500 µm. (C,D) Uninjured caudal peduncle sites showing the intermuscular bones (arrows). Epineurals are dorsally located and epipleurals are ventrally located. (E) Injured caudal peduncle site, 1 month later, showing hypertrophied hypurals and parahypural (arrows). (F) Injury site showing hypertrophied intermuscular bones (arrows). (G) Uninjured thoracic region showing normal epineural intermuscular bones (arrows). The absence of epipleurals in the thoracic region can be noted. (H,I) Injured thoracic region, 1 month later, showing hypertrophied intermuscular bones (arrows). (J) Hypertrophied neural spines in an injured fish (arrows). (K) Comparison of intermuscular bone size between the uninjured and injured side (*n*=12 bones/group from four fish), with significantly larger intermuscular bones observed on the injured side (*y*-axis is in μm²). Boxes show the interquartile range, whiskers show the highest and lowest value, and the median is marked with a line. ***P<0.0001 (two-tailed unpaired *t*-test).

Intermuscular bones form an integral part of the skeletal system in teleost fish. They are located in the myosepta on both sides of the vertebrae and considered to develop via intramembranous ossification of myoseptal tendons without undergoing a cartilaginous phase (Li et al., 2021; Nie et al., 2019). In most teleost fish, there are three sets of intermuscular bones: epineurals, epicentrals and epipleurals, attaching ligamentously to neural, central and hemal arches or ribs, respectively (Nie et al., 2019). In the zebrafish, however, only epineural and epipleural bones are present (Fig. 2C,D,G), the former in each myoseptum and the latter only in myosepta caudal to the ribs throughout the tail region (Bird and Mabee, 2003; Yao et al., 2015). Their function is to transmit force between muscle segments and to increase the stiffness of the body. During development of the zebrafish, these bones ossify from the distal to the proximal end, and the ossification is believed to be influenced by the mechanical load induced by swimming (Yao et al., 2015).

Following caudal peduncle contusion injuries (Fig. 2A), we observed a larger-than-normal size in the hypurals, parahypural, as well as the corresponding haemal and neural spines (Fig. 2E). The increase in bone size was evident when comparing them to the typical appearance of these bones in control fish of the same age and size (Fig. 2C). Furthermore, we observed that the intermuscular bones located at the injury site, specifically the distal-most and pre-distal pairs, which are entirely encased within muscle tissue and not connected to the axial spine, displayed hypertrophy in the majority of the injured fish (Fig. 2F). This observation raises the possibility that the muscle contusion alone could trigger factors conducive to bone growth.

To examine a similar occurrence at an alternate, non-mobile site, we induced recurring unilateral contusions by gentle stroking using a blunt tip K-pin on the dorsal thoracic region (Fig. 2A,B), designating the uninjured side as the control (Fig. 2G). We observed strikingly large intermuscular bones on the contused side in all the injured fish (Fig. 2H,I). In addition, some of the fish demonstrated hypertrophy of the neural spines in the region (Fig. 2J). This observation confirms that the contusion site favours bone growth. To quantify the changes, we outlined the bones and measured the contained area. This process was repeated for the corresponding uninjured side, and the extent of hypertrophy was determined by comparing the bones on either side. It was evident that the intermuscular bones were significantly larger on the injured side (57,731.8±18,801.4 μm², indicated as the mean±standard deviation (±s.d.) compared to those on the uninjured side (13,807.01±3385.35 μm²), by more than four-fold (4.18; *P*<0.0001) (Fig. 2K). Given that osteo-induction and subsequent growth is required for development of any bone, the zebrafish injury microenvironment harbouring these properties following injury can serve as a valuable tool to study heterotopic bone formation. We investigated the feasibility of a longitudinal study assessing images of intermuscular bones before and after injury using live adult transparent *casper* mutants stained with Alizarin Red. However, owing to the deep location of the intermuscular bones, they were not clearly visualised from the outside (Fig. S2).

### Identification of a highly penetrant model for heterotopic bone involving the pectoral fin

**Fig. 3.**
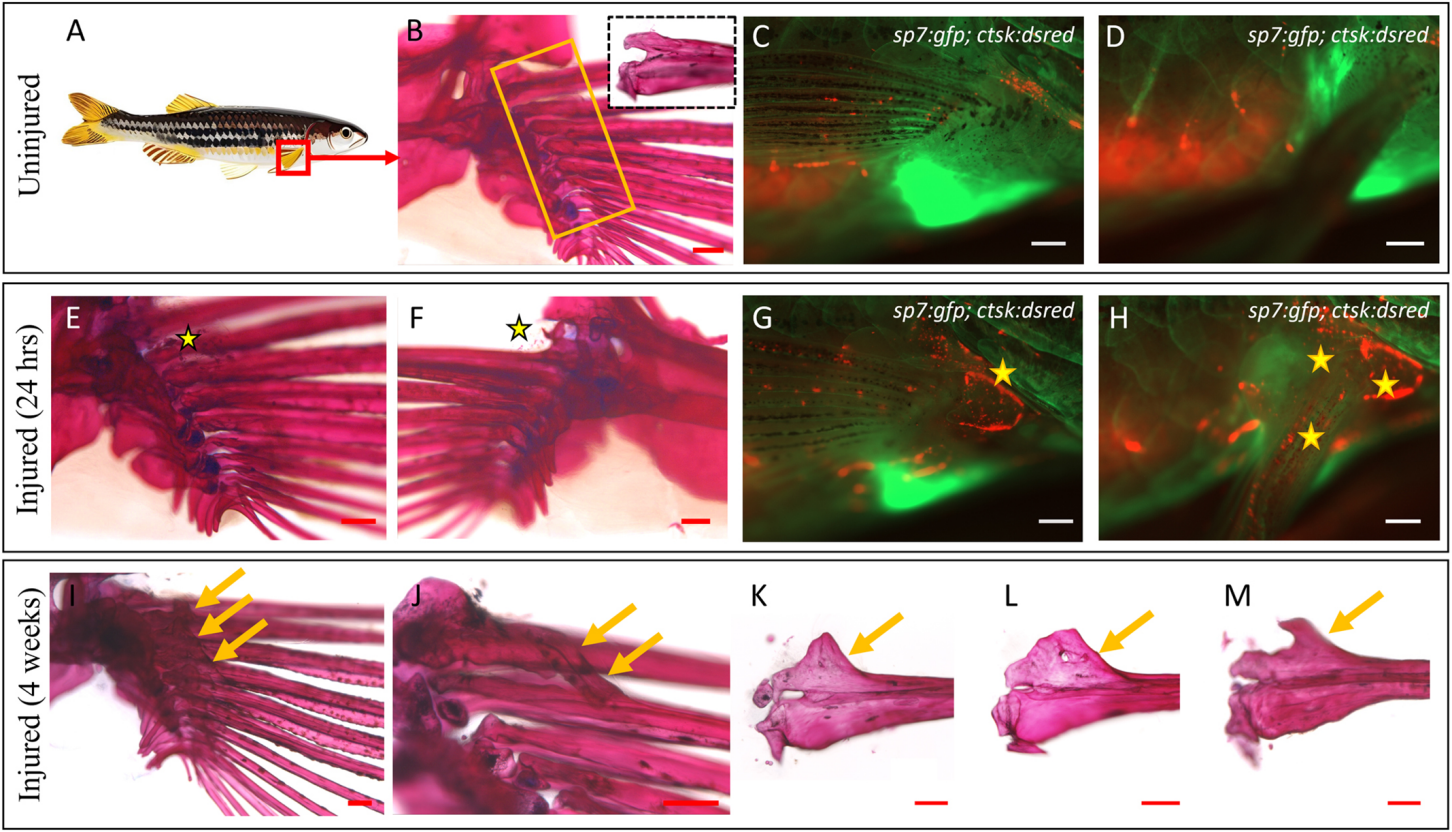
**Consistent heterotopic ossification in the form of spurs and bridges in the pectoral fin.** (A) Graphic depicting the injury site. (B) Medial view of an uninjured right pectoral fin stained with Alizarin Red. The yellow box indicates the injury site; the zoomed-in dotted box focuses on the articular region of a dissected fin ray, comprising the dorsal and ventral hemirays. (C) Lateral view of an uninjured pectoral fin of *Tg(sp7:gfp; ctsk:dsred)* fish under fluorescence microscopy. (D) The same fish as in C after elevating the pectoral fin to show the inner (medial) side where the absence of osteoclastic activity can be noted. (E,F) Medial and lateral views of an injured pectoral fin, 24 h post injury, showing tiny flecks of bone (stars) arising from the microfractures. (G) Lateral aspect of an injured pectoral fin of *Tg(sp7:gfp; ctsk:dsred)* fish showing osteoclastic activity (star) at 24 h post injury. (H) Osteoclastic activity on the medial aspect seen after elevating the fin (stars). (I) Injured pectoral fin, 1 month after injury. Arrows highlight the heterotopic bone spurs. (J) Heterotopic bridging bone noticed between the marginal ray and the second ray in one of the injured fish (arrows). (K-M) Different forms of heterotopic bone spurs encountered following injury (arrows). Images are representative of the first batch of five fish per condition. The observed heterotopic ossification phenotype was consistently reproducible in subsequent batches (*n*=32 fish). Scale bars: 200 µm (B,E,F,I-M); 500 µm (C,D,G,H).

The medial aspect of the pectoral fin was selected as an alternative site due to its mechanistic relevance (Fig. 3A,B). In this region, the bulk of muscles responsible for fin movements, notably the adductor superficialis and the adductor profundus, envelop the scapulo-radialis bones and the proximal section of the fin rays (Siomava and Diogo, 2018). In addition, the fin rays at the pectoral fin are bulkier and stronger than those at the tail fin (Fig. 3B). Here, we induced heterotopic bone by creating injuries involving both muscle and bone near the articulation of the pectoral fin with the radial bones, precisely where the dorsal hemi-ray and ventral hemi-ray fuse (Fig. 3B).

The fish were anaesthetised and the pectoral fins were injured as described in the Materials and Methods using a Dumont #5 forceps. Injury to the medial muscle bulk and microscopic damage to the proximal aspect of the pectoral fin rays were observed post injury (Fig. 3E,F). Injuries were repeated using the same technique twice at 48-h intervals to sustain inflammation. Live imaging of this region posed challenges because the body of the fish beneath the pectoral fin obstructs the optical plane. However, using double-transgenic *Tg(sp7:gfp;ctsk:dsred)* fish, we identified early osteoclastic activity in response to the damage (Fig. 3C,D,G,H). All fish exhibited heterotopic bone formation, manifesting as spurs and/or bridges between the rays (*n*=5, Fig. 3I-M). This penetrance was consistently reproducible in subsequent batches of fish with distinct heterotopic bone formation observed in all the injured fish (*n*=32). Micro-computer tomography (CT)-based volumetric assessment comparing the uninjured and injured sides was not conducted. Nevertheless, as generating these injuries is straightforward and the formation of the pathology was fully penetrant, this model is easily scalable and reproducible for studying heterotopic ossification.

### Bulk RNA sequencing for transcriptional profiling of injured tissue

**Fig. 4.**
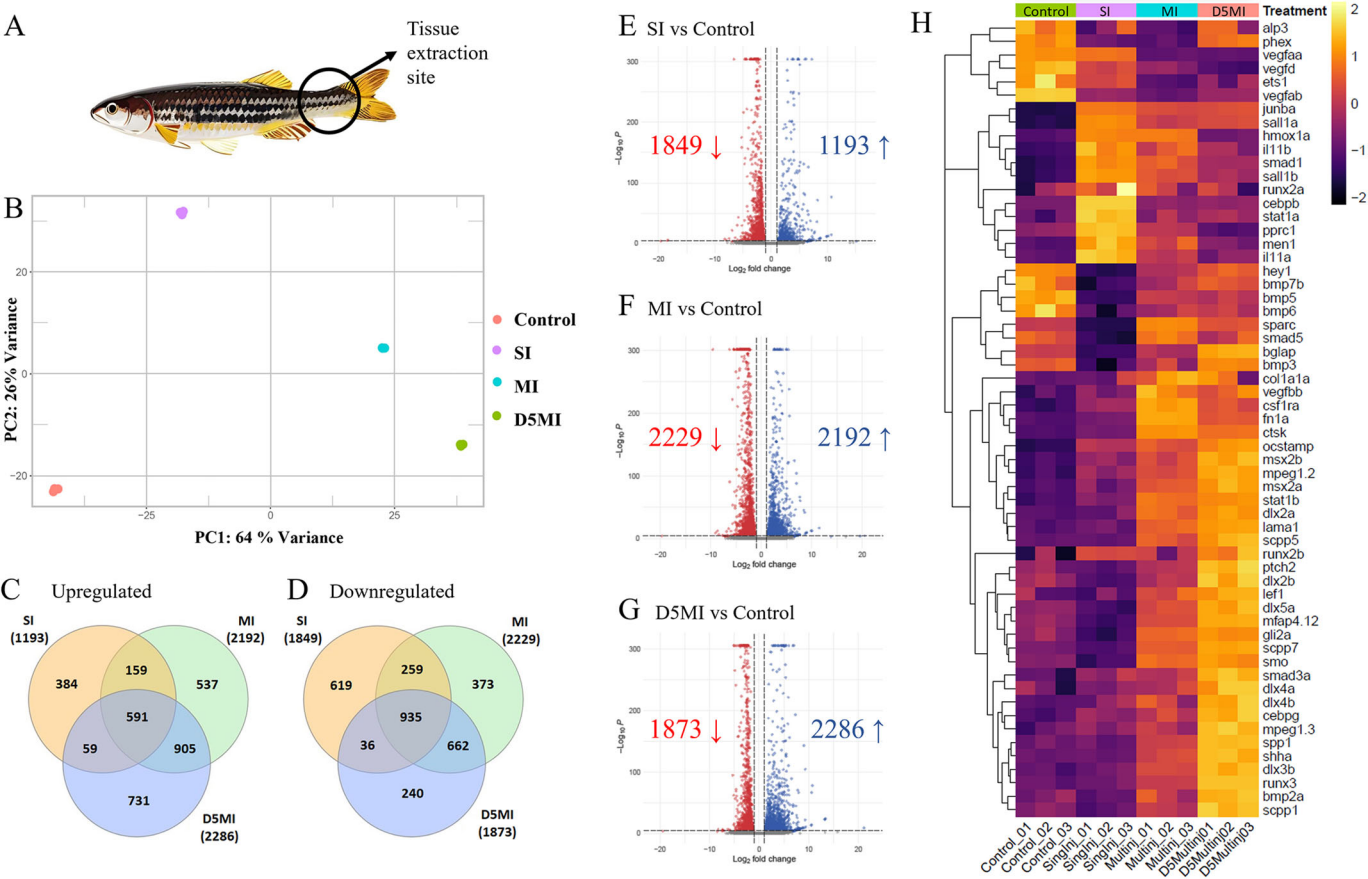
**RNA-sequencing cluster analysis and differential gene expression.** (A) Graphic showing the caudal peduncle contusion site from where tissues were extracted. (B) Principal component analysis (PCA) plot displaying sample distances. Control, no injury; SI, 24 h after single injury; MI, 24 h after multiple injuries; D5MI, 5 days after multiple injuries. *n*=9 fish per condition; *n*=3 fish per biological replicate. (C,D) Venn diagrams illustrating the number of upregulated and downregulated genes in each comparison. (E-G) Volcano plots depicting the overall numbers of upregulated and downregulated genes among the three comparisons, with log_2_(fold change) on the *x*-axis and significance on the *y*-axis. (H) Heat map of selected genes of interest.

To investigate the transcriptional response underlying the observed enlargement of bones at the contusion site, we conducted genome-wide bulk RNA sequencing on contused muscle from the caudal peduncle region, as this region allows for easy harvesting of the entire muscle bulk (Fig. 4A). Investigation was not solely focused on comparing uninjured and injured conditions, but rather assessed the response characteristics in the case of multiple recurring injuries. Contused muscle tissue was dissected at four different time points: no injury (control), 24 h after single injury (SI), 24 h after multiple (three) injuries (MI) and 5 days after multiple (three) injuries (D5MI). Principal component analysis (PCA) revealed similarity between replicates and differences across conditions (Fig. 4B). Further analysis focused on three comparisons: (1) SI versus control, (2) MI versus control and (3) D5MI versus control, where Benjamini–Hochberg-adjusted *P*-values below 0.05 and log_2_(fold change or FC) of 1.5 were considered significant for determining differentially expressed genes.

The numbers of upregulated and downregulated genes depicted a combination of both unique and overlapping signatures per condition (Fig. 4C,D). Pairwise comparisons revealed 1193 upregulated and 1849 downregulated genes for the SI condition, 2192 upregulated and 2229 downregulated genes for the MI condition, and 2286 upregulated and 1873 downregulated genes for the D5MI condition (Fig. 4E-G). Assessing the top 10,000 differentially expressed genes to understand the global transcriptomic changes revealed a temporal relationship, with the expression profiles of the D5MI group more closely related to those of the MI group compared to those of the SI and control groups (Fig. S3). Analysis of the differentially expressed genes (Table S1-S3), Gene Ontology (GO) classification and pathway enrichment analysis (KEGG) suggested that the immune response characterised by apoptosis and upregulation of inflammatory mediators is triggered after muscle contusion injury (Fig. S4-S6). As a result of recurring injuries, this immune signature persisted. Specifically, subsequent to an initial upregulation of the genes *il11a* and *il11b*, an elevation in various osteoblast differentiation markers was observed over time. These included *runx3, bglap, dlx2a, dlx2b, dlx3b, dlx4a, dlx5a, msx2a, spp1, fn1a* and the teleost-specific genes *scpp1*, *scpp5* and *scpp7* (Huang et al., 2007; Kawasaki, 2009). In addition, developmental patterning genes such *sall1a*, *sall1b*, *hoxa13a* and *hoxa13b* (Perez et al., 2010) were upregulated. Interestingly, *kcnk5b*, which acts locally within the mesenchyme of fins and barbels to specify appendage size (Perathoner et al., 2014), and the osteoclast-specific genes *stat1b*, *ocstamp*, *csf1ra* and *ctsk* were also upregulated, though at the later time point (D5MI) (Fig. 4H). These notable changes could underlie the growth and enlargement of bones after injury.

### The role of potassium channel activity in regulating response characteristics of heterotopic bone formation

**Fig. 5.**
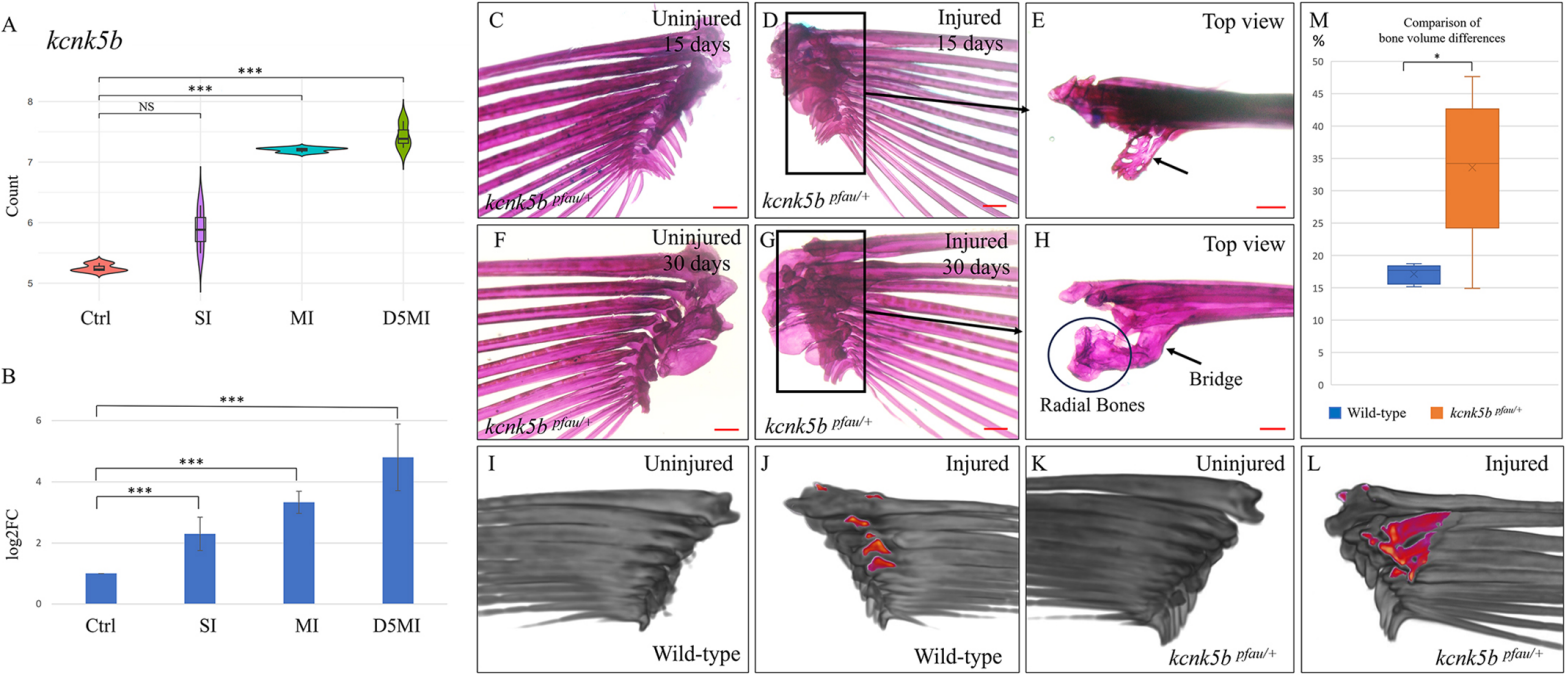
**Differential expression of *kcnk5b* and higher magnitude of heterotopic bone formation in *kcnk5b^pfau/+^* mutants.** (A) Violin plot depicting the differential expression of *kcnk5b* as inferred from RNA sequencing. The *y*-axis represents log2(normalised count). Control, no injury; SI, 24 h after single injury; MI, 24 h after multiple injuries; D5MI, 5 days after multiple injuries. *n*=9 fish per condition; *n*=3 fish per biological replicate. (B) qRT-PCR validation of the differential expression of *kcnk5b*. *n*=9 fish per condition; *n*=3 fish per biological replicate. (C,D) Uninjured left and injured right pectoral fins of a *kcnk5b^pfau/+^* mutant zebrafish visualised by Alizarin Red staining 2 weeks post injury. (D) Medial aspect of the injured pectoral fin showing extensive heterotopic ossification (boxed area). (E) Top view of the injured pectoral fin shown in D clearly illustrating the extent of heterotopic bone (arrow). (F,G) Uninjured left and injured right pectoral fins of a *kcnk5b^pfau/+^* mutant zebrafish visualised 1 month post injury. (G) Medial aspect of the injured pectoral fin showing extensive heterotopic ossification (boxed area). (H) Top view of the injured pectoral fin shown in G indicating bridging (arrow) between the second fin ray and the radial bones. (I-L) 3D-reconstructed CT scans. (I,J) Medial view of the uninjured left and injured right pectoral fins of a wild-type fish. (J) Injured pectoral fin showing heterotopic bone spurs highlighted in colour. (K,L) Medial view of the uninjured left and injured right pectoral fins of a *kcnk5b^pfau/+^* mutant fish. (L) Injured pectoral fin showing extensive heterotopic bone (highlighted in colour), unlike that of wild-type fish. (M) Box and whisker plot showing the comparison of bone volume differences at 4 weeks post injury (*n*=5 fish per group). The blue box represents the difference between bone volume of uninjured (control) and injured wild-type fins, expressed as percentages. The orange box represents the same for *kcnk5b^pfau/+^* mutants. Note the significant positive difference in *kcnk5b^pfau/+^* mutants. Boxes show the interquartile range, whiskers show the highest and lowest value, and the median is marked with a line. NS, not significant; *P<0.05; ***P<0.001 (Wilcoxon rank-sum test with Benjamini-Hochberg correction in A; two-tailed unpaired *t*-test in B, M). Scale bars: 200 µm (C-H).

It has been shown that elevated activity of potassium channels, such as Kcnk5b, can influence skeletal growth and patterning in zebrafish (Perathoner et al., 2014). These channels are crucial for regulating the cellular electrical potential and are involved in diverse biological processes, but their precise activity in tissues is not well characterised or understood. We examined *kcnk5b* expression in response to contusion injuries in our transcriptome data and observed a progressive and significant increase over time (Fig. 5A). This observation was further validated through quantitative real-time PCR (qRT-PCR) analysis (Fig. 5B). Consequently, we were interested in exploring the heterotopic bone formation response in the context of altered Kcnk5b activity. The *pfau* gain-of-function mutant of Kcnk5b (*kcnk5b^dt30mh/+^*) displays elongated fin segments due to increased growth rate of lepidotrichia (Perathoner et al., 2014). Using this mutant in our assay, we observed an accelerated response in injured fish (*n*=4), with detectable heterotopic bone formation occurring in just 2 weeks after the last injury, in contrast to the response of wild-type fish (Fig. 5C-E). In a separate batch, 1 month after the injury, all affected pectoral fins (*n*=5) exhibited markedly greater heterotopic bone formation compared to those of their wild-type counterparts (Fig. 5F-H). Notably, in one of the injured pectoral fins, we observed bridging between the fin rays and the radial bones (Fig. 5H), emphasizing the extensive nature of the heterotopic response. To quantify these findings, we stained the fins with 1% silver nitrate and performed micro-CT scans (Fig. 5I-L) (Charles et al., 2017). Using the 3D Slicer image computing platform, we compared the bone volumes of the injured and uninjured sides in all the fish. Our findings revealed that the injured fins of *kcnk5b^pfau/+^* mutants, in contrast to those of wild-type fish, exhibited a significantly higher bone volume (*P*=0.02) compared to the bone volume of fins on the uninjured side as early as 2 weeks following injuries (Movies 1 and 2). Furthermore, after 1 month, although the wild-type fish showed only a 17.1±1.5% (mean±s.d.) higher bone volume on the injured fin compared to that of the uninjured side, the *kcnk5b^pfau/+^* mutants displayed a significantly greater difference of 33.6±11.9% (*P*=0.01) (Fig. 5M). Therefore, it appears likely that potassium channel signalling, specifically via Kcnk5b, plays a crucial role in both initiation and manifestation of the heterotopic bone formation response.

### Role of Il-11 signalling in the development of heterotopic ossification

**Fig. 6.**
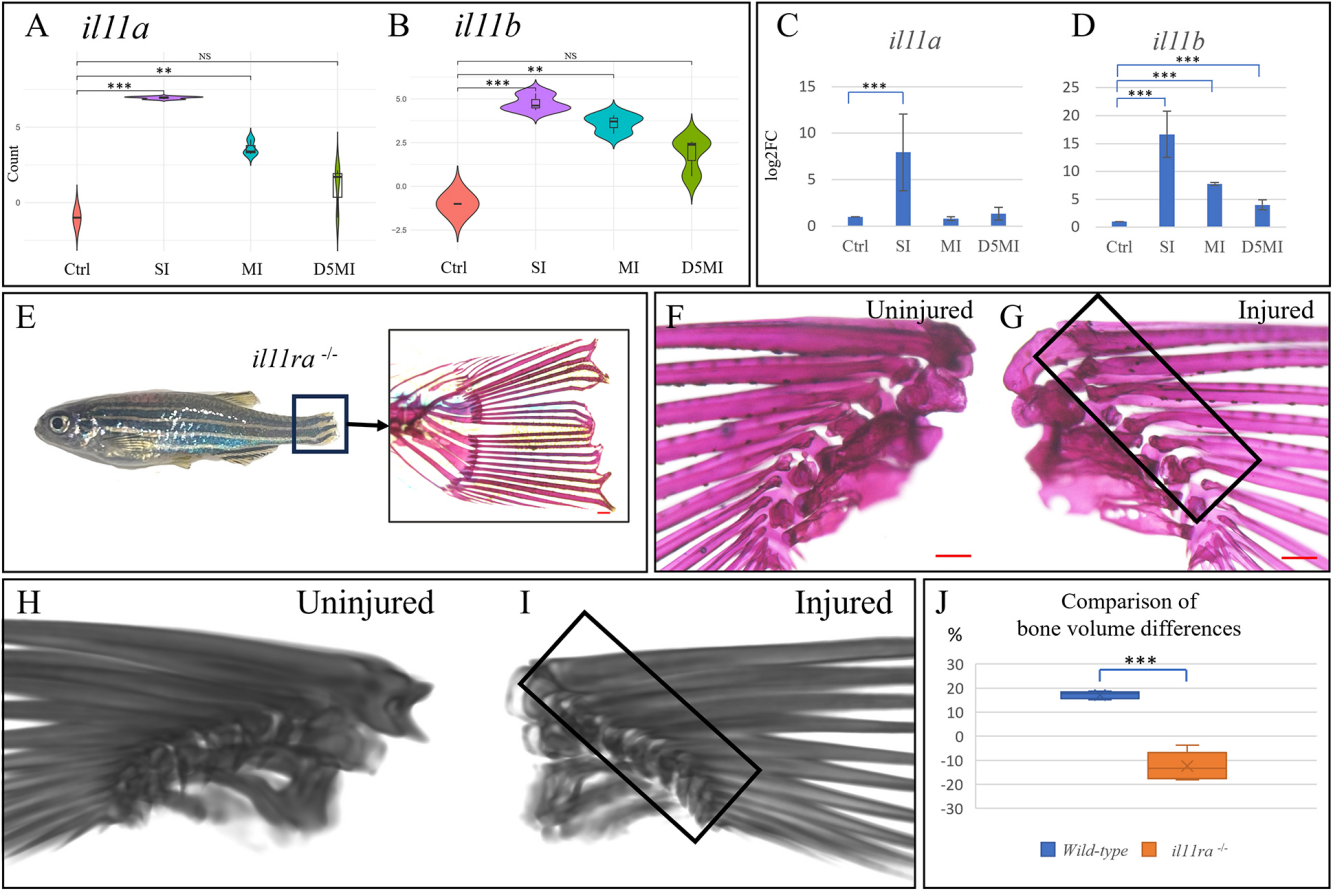
**Differential expression of genes encoding Il-11, and absent heterotopic ossification in *il11ra^−/−^* mutants.** (A,B) Violin plots depicting the differential expression of *il11a* and *il11b* as per RNA sequencing. The *y*-axis represents log2(normalised count). Control, no injury; SI, 24 h after single injury; MI, 24 h after multiple injuries; D5MI, 5 days after multiple injuries. *n*=9 fish per condition; *n*=3 fish per biological replicate. (C,D) qRT-PCR validation of the differential expression of *il11a* and *il11b*. *n*=9 fish per condition; *n*=3 fish per biological replicate. (E) *il11ra*^−/−^ mutant zebrafish demonstrating impaired tail fin regeneration (boxed area) at 2 weeks following injury. The zoomed-in image shows an Alizarin Red-stained section of a non-regenerated tail fin. (F,G) Uninjured left and injured right pectoral fins of an *il11ra*^−/−^ mutant zebrafish visualised 1 month post injury. (G) Medial view of the injured right pectoral fin showing no signs of heterotopic bone. (H,I) Three-dimensional reconstructed computed tomography scans. (I) Medial view of the injured right pectoral fin showing no heterotopic bone. (J) Box and whisker plot showing the comparison of bone volume differences (*n*=5 fish per group). The blue box represents the difference between bone volume of uninjured (control) and injured wild-type fins, expressed as percentages. The orange box represents the same for *il11ra*^−/−^ mutants. Note the significant negative difference in *il11ra*^−/−^ mutants. Boxes show the interquartile range, whiskers show the highest and lowest value, and the median is marked with a line. NS, not significant; **P<0.01; ***P<0.001 (Wilcoxon rank-sum test with Benjamini-Hochberg correction in A,B; two-tailed unpaired *t*-test in C,D,J). Scale bars: 200 µm (E-G).

Expression of *il11a* and *il11b* was significantly upregulated as an early response to contusion injuries, with the levels of both transcripts gradually declining at later time points (Fig. 6A,B). This observation was validated through qRT-PCR analysis (Fig. 6C,D). Previous studies revealed that Il-11-encoding genes (*il11a* and *il11b*) exhibited the most significant induction and highest expression after tissue damage in the zebrafish cardiac ventricle and caudal fins (Allanki et al., 2021). Moreover, transcriptome data from various regenerating tissues in zebrafish, African killifish, lungfish, *Xenopus* and axolotl have demonstrated an evolutionarily conserved and injury-responsive induction of Il-11 (Darnet et al., 2019; Fang et al., 2013; Gerber et al., 2018; Tsujioka et al., 2017; Wang et al., 2020). In the case of zebrafish, *il11ra^bns251^* mutants, lacking the Il-11 receptor, similar to *Il11r* mutant mice, survive into adulthood without noticeable developmental defects (Allanki et al., 2021). However, they exhibit impaired tail fin regeneration following fin clipping (Fig. 6E). Upon injuring these mutants (*n*=5), it was observed that the *il11ra*^−/−^ mutants failed to exhibit heterotopic bone formation similar to that seen in their wild-type counterparts (Fig. 6F-I). Notably, although wild-type siblings exhibited higher bone volume on the injured pectoral fin compared to that on the uninjured side, the *il11ra*^−/−^ mutants demonstrated a significantly lower bone volume (−12.4±5.9%; *P*<0.0001) in the injured fins (Fig. 6J; Movies 3 and 4). This difference was evident both visually and in volumetric assessments using micro-CT scans. Although we noticed a single spur formation in one of the injured *il11ra*^−/−^ mutants, the overall bone volume in the injured fin was lower compared to that in the uninjured side. These observations emphasise the pivotal role of Il-11 signalling in the normal damage response program. Furthermore, they suggest that targeting of Il-11 function and its suppression have implications for modulating injury-induced inflammation and the subsequent development of heterotopic bone.

### Intermuscular bone hypertrophy in Kcnk5b gain-of-function and Il11ra loss-of-function mutants

**Fig. 7.**
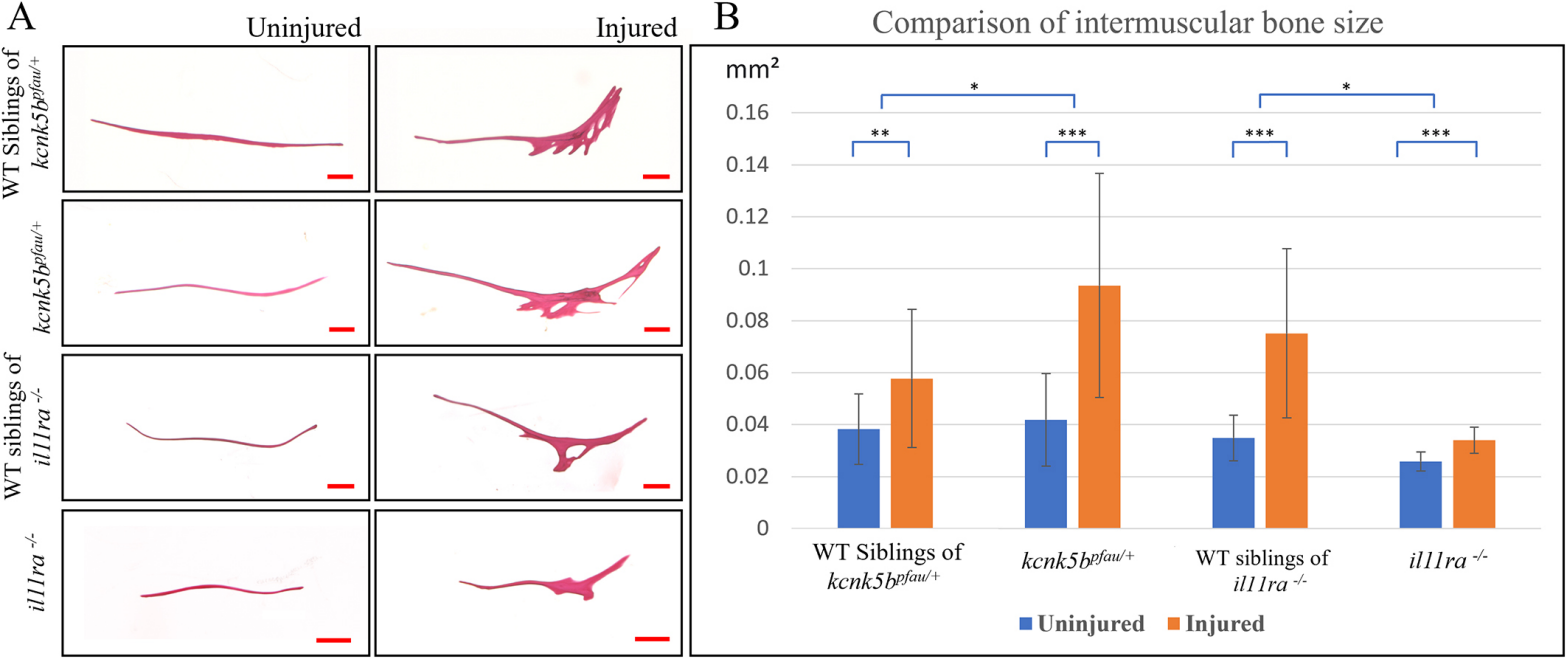
**Analysis of thoracic intermuscular bone size following injury in *kcnk5b^pfau/+^* and *il11ra^−/−^* mutants.** (A) Representative images comparing the thoracic intermuscular bones on the uninjured and injured sides in *kcnk5b^pfau/+^* (*n*=24 bones/side from five fish) and its wild-type (WT) sibling controls (*n*=18 bones/side from five fish), as well as in *il11ra^−/−^* (*n*=14 bones/side from four fish) and its WT sibling controls (*n*=14 bones/side from four fish). Scale bars: 200 µm. (B) Quantitative analysis revealed that the difference in size between uninjured and injured fins was more pronounced in the *kcnk5b^pfau/+^* mutants compared to that in their wild-type siblings. In contrast, the difference was minimal in *il11ra*^−/−^ mutants. Data show the mean±s.d. *P<0.05; **P<0.01; ***P<0.001 (two-tailed unpaired *t*-test for parametric data comparison; Mann-Whitney U test for non-parametric data comparison).

Given the heterotopic bone formation responses observed in fish carrying either the Kcnk5b gain-of-function mutation or the Il11ra loss-of-function mutation, we explored the development of intermuscular bone hypertrophy resulting from thoracic contusions in both mutants. We assessed intermuscular bone hypertrophy response conducted in a series of three injury episodes, each separated by a 48-h interval as demonstrated previously, using Alizarin Red staining. Our findings revealed that the intermuscular bones were significantly larger on the injured side compared to those on the uninjured (control) side in all fish, regardless of their mutation status (Fig. 7A). We then calculated the difference in the size of intermuscular bones between the injured and uninjured sides, revealing distinct responses between the mutants and wild-type fish. Specifically, fish carrying the Kcnk5b gain-of-function mutation exhibited a greater difference compared to wild-types, highlighting a significant response to the injury (Fig. 7B). In contrast, fish homozygous for the Il11ra loss-of-function mutation displayed a limited response. These findings provide valuable insights into the role of these mutations in the response to thoracic contusions and subsequent intermuscular bone hypertrophy.

## DISCUSSION

We have established what is, to our knowledge, the first zebrafish model of injury-induced heterotopic ossification that closely resembles human MOT. The injuries carried out in this study were designed to be akin to the types of injuries occurring in humans, such as contusions and microtrauma to bone, which have the potential to lead to heterotopic ossification. In contrast to previous models in mammals (Cao et al., 2023; Dey et al., 2017; Epperson et al., 2021; Williams et al., 2019), these injuries in zebrafish do not necessitate a sophisticated experimental setup. Furthermore, the formation of mature heterotopic bone is rapid, occurring within a range of 3 to 4 weeks. The presentation of heterotopic bone also mimics numerous instances of MOT reported in the literature, where the heterotopic bone frequently originates from the underlying bone and occupies the adjacent soft tissue area. Additionally, the bridging heterotopic ossification observed between two underlying bones in some of the injured fish mimics extensive cases of heterotopic bone formation observed in patients with FOP and in some reported cases of MOT (Kanthimathi et al., 2014; Kotb et al., 2023).

There is a well-entrenched grading system for heterotopic ossification occurring after hip replacement/arthroplasty surgeries, known as the Brooker classification of heterotopic ossification (Brooker et al., 1973). According to this system, there are four grades of heterotopic ossification that can occur following hip replacement: grade 1 – islands of bone in the soft tissues; grade 2 – bone spurs, >1 cm gap between opposing bony surfaces; grade 3 – bone spurs, <1 cm gap between the opposing surfaces; and grade 4 – complete ankylosis (Brooker et al., 1973). Examining the appearance of heterotopic bone evoked in zebrafish, we correlated the responses with this classification system: the formation of spurs aligned with grades 2 and 3, and complete bridging aligned with the most severe grade 4. In addition, aligning the responses with the frequency in humans (Di Benedetto et al., 2019), spurs representing the milder grades were prevalent, whereas bridges representing grade 4 were notably less frequent. This could be attributed to mechanical factors, a long-established concept that remains valid to this day (Makins, 1924). Injury to the bones or periosteum causes blood extravasation, resulting in the formation of tracks within the soft tissues (Zheng et al., 2024), particularly when a muscle tear is present. This allows osteoblasts and progenitor cells to migrate into these tracks, creating an optimal environment for their preservation, differentiation and proliferation (Zhang et al., 2022). The size and extent of heterotopic ossification are influenced by the limiting boundaries of adjacent soft tissues.

Drawing parallels between our zebrafish model of pectoral fin heterotopic ossification and hip arthroplasty-induced heterotopic ossification, both involve the creation of raw areas on the bone (Moretti and Post, 2017). During hip arthroplasty, raw surfaces on the bones are generated both at the femoral end and at the acetabular end. Coincidently, this is where heterotopic bone spurs often form. In the zebrafish pectoral fin post injury, microscopic damage to the bone was evidenced, leaving behind small fragments of bone in the soft tissue, reminiscent of the reaming debris in human surgery. Although our analysis suggests that this debris was resorbed by increased osteoclast activity, it resulted in a raw area on the surface of the bone where heterotopic ossification developed. This phenomenon can also be related to femoral intramedullary nailing, a common surgical procedure to stabilise femoral shaft fractures in humans (White et al., 2011). Generally, there are no issues; however, in rare circumstances, heterotopic ossification may be observed at the entry site, typically manifesting as bony outgrowths attached to the underlying bone (Botolin et al., 2013; Marks et al., 1988). Despite the skeletal structures in the zebrafish being neither homologous nor of orthologous developmental origin (endoskeletal versus intramembranous) to the human bone, these reports consistent with our model indicate that raw surfaces on the bone, even in the absence of a complete fracture, have the potential to form heterotopic bone under appropriate conditions.

The occurrence of heterotopic ossification varies in its severity and location across different types of traumas (Meyers et al., 2019). It manifests in approximately 30% of patients following fractures or dislocations in the elbow (Hong et al., 2015). High-energy extremity trauma, traumatic brain injury or spinal cord injury, and other neurological disorders are reported to increase this incidence to over 50% (Forsberg et al., 2009). The highest reported incidence is associated with severe traumatic amputations, exceeding 90% (Daniels et al., 2018), and the lowest reported incidence is linked to burn injuries, ranging from 3.5 to 5.6% (Hu et al., 2021). In our model, the incidence of heterotopic ossification due to contusion injury at the caudal peduncle region was 26%. Even though this aligns with the incidence of heterotopic ossification in humans, it does not provide the needed penetrance for an efficient experimental model, as is also the case with some of the previously proposed animal models of trauma-induced heterotopic ossification (Anthonissen et al., 2014; Walton and Rothwell, 1983). Alternatively, the pectoral fin following injuries demonstrated 100% penetrance, as did the intermuscular bone hypertrophy resulting from muscle contusions. Consequently, these models are suitable for scaling up to facilitate extensive experimental analysis of management strategies for heterotopic ossification disorder.

Data from patients with FOP and *in vivo* animal models of FOP suggest that the inflammatory microenvironment harbours mesenchymal stem cells (MSCs) that ultimately differentiate into osteoblasts leading to new bone formation (Billings et al., 2008; Kaplan et al., 2011). Several studies have sought to determine the source of these MSCs. Initially, cells displaying disrupted BMP signalling and abnormal osteogenic differentiation were believed to be from the myogenic lineage (Katagiri et al., 2018); however, further investigations suggested the local MSC population at the site of inflammation to be a more relevant source of progenitor cells that differentiate into chondrocytes and osteoblasts (Billings et al., 2008). Furthermore, various sources such as the local stromal/fibroblastic cells, endothelial cells through the endothelial-mesenchymal transition, Scx^+^ tendon progenitor cells, bone marrow-derived muscle-resident Mx1^+^ cells, glutamate transporter (Glast or SLC1A3)-expressing progenitor cells and certain circulating osteogenic precursor cells with access to bone-forming sites are linked to the origin of these MSCs (Dey et al., 2016; Pignolo and Kassem, 2011; Pulik et al., 2020; Ranganathan et al., 2015; Wosczyna et al., 2012). Nevertheless, this continues to be an area of ongoing investigation. Considering these mechanisms, inhibiting osteoblastic differentiation may appear to be a feasible therapeutic approach. However, it cannot be executed in situations where there is a fracture, as doing so would affect fracture healing. Hence, there is a need for alternative targets.

Our data on the transcriptional response to trauma revealed gene expression signatures that have not previously been explored within the context of heterotopic ossification. There is a paucity of published transcriptome datasets for contused muscle tissue, particularly those examining repeated injuries at multiple time points as in this study. However, Ren et al. (2021) conducted a study involving one-time muscle injuries induced by dropping a 500 g weight from a height of 50 cm onto the right hindlimb of rats through a free-fall motion. Rats from the experimental groups were sacrificed at intervals of 4, 8, 12, 16, 20, 24 or 48 h after the injury, and RNA sequencing was carried out on the extracted injured tissues. Our observations align with the findings of Ren et al. (2021), specifically when examining the outlined GO terminologies. Notably, transcripts associated with biological processes such as response to stimuli, oxidative stress, inflammation, infiltrating immune cells, apoptosis, haematopoiesis and microtubule processes show similarity, especially during the early time points. In addition, our dataset displayed comparable enrichment in KEGG pathways associated with the immune response, cascade reactions, apoptosis and phagocytosis, as well as repair-related processes.

Given that the caudal peduncle contusion site exhibited heterotopic ossification at a limited frequency, it showed a more pronounced hypertrophy of intermuscular bones following injury. Notwithstanding the varied expressivity, the RNA sequencing findings closely corresponded to the changes causing growth and enlargement of intermuscular bones. Although we did not spatially characterise the expression patterns, our findings shed light on the differentially regulated genes at contusion sites with the potential to promote osteogenesis, despite the absence of bony injuries. Although the master switch governing this process remains to be defined, these genes and pathways can be designated as targets to be studied using genetic manipulations and pharmacological interventions to determine whether they exert any inhibitory effect on the heterotopic bone formation response.

We first focused our analysis on the observed differential expression of Kcnk5b, a two-pore potassium-leak channel that regulates membrane potential by the outward flow of potassium from the cell (Goldstein et al., 2001). Although the exact mechanisms through which these ion channels modulate various pathways are not yet fully understood, their relationship is apparent. Mutations in the Kir2.1 (KCNJ2) potassium channel have been linked to Andersen–Tawil Syndrome (Ozekin et al., 2020; Plaster et al., 2001; Yoon et al., 2006). Kir2.1 plays a crucial role in BMP signalling, and genetic disruptions in this channel result in reduced activation of downstream BMP targets (Belus et al., 2018). This is linked to the modulation of BMP release via the regulation of membrane potential and the levels of intracellular calcium. In mice, genetic knockout of Kir2.1 mirrors the phenotypes seen in BMP2/4 mutants, which include the development of severe craniofacial features such as an enlarged fontanelle, underdeveloped mandible, nasal bone hypoplasia and cleft palate, along with limb and digit abnormalities (Bonilla-Claudio et al., 2012; Ozekin et al., 2020; Sacco et al., 2015; Suzuki et al., 2009). Similarly, disruptions in the TASK3 potassium channel, also known as KCNK9, have been linked to conditions such as scoliosis, cleft palate and distinctive facial features in humans (Barel et al., 2008). This underscores the importance of the precise regulation of cellular membrane potential for the accurate formation of specific structures.

Consistent with the above findings, the long finned mutants in zebrafish (*lof*, *alf* and *schl*) provide additional evidence of the vital significance of potassium channel conductance and electrophysiological signals in establishing accurate body-to-appendage proportions (Lanni et al., 2019; Perathoner et al., 2014; Silic et al., 2020; Stewart et al., 2021). Although the mechanisms mentioned above highlight the involvement of potassium channels in regulating zebrafish fin development, our transcriptomic analysis revealed a progressive increase in the expression of *kcnk5b* over time following muscle contusion injury, indicating its role in natural healing. This discovery, along with the increased magnitude of heterotopic bone formation observed after pectoral fin injuries in the Kcnk5b gain-of-function mutants, strongly points towards a potential role for Kcnk5b in regeneration following injury, influencing the extent of new bone formation. This not only opens up new avenues for investigating the interplay between potassium channels and the pathways governing regeneration, but, as these channels are often targets of small-molecule drugs, its action also provides a basis for therapeutic targeting of MOT – one that is titratable.

We next investigated processes sufficient to promote heterotopic bone growth. Upon injury, regenerative inflammation promotes tissue repair by a timed and coordinated infiltration of diverse cell types and the secretion of growth factor, cytokine and lipid mediators (Caballero-Sanchez et al., 2022). One of the key aspects of this process is the release of interleukins, especially the Il-6-type cytokines that act locally and systemically to generate a variety of physiologic responses (Jawa et al., 2011). In multiple transcriptome datasets from regenerating tissues, including those from zebrafish, Il-11 has been demonstrated to be induced as a response to injury. We also noticed high expression of both paralogous genes encoding Il-11 (*il11a* and *il11b*) after injury. Studies have shown the upregulation of Il-11 following fractures and its role in promoting regeneration (Kidd et al., 2010). Additionally, transgenic mice overexpressing human IL-11 experience increased bone formation without an effect on bone resorption or osteoclastogenesis (Kidd et al., 2010), due to its enhancing the effect of BMP2 and inhibiting bone marrow adipogenesis (Takeuchi et al., 2002). These observations indicate that Il-11 promotes osteoblastic activity at injury sites without influencing osteoclastic activity. This could clarify the decrease in bone volume observed in the injured fin of *il11ra^−/−^* mutants, potentially due to bone resorption by osteoclasts following the microfractures while the osteoblastic activity is impaired. Nevertheless, this discovery highlights a potential new path for intervention, through broadly targeting the inflammatory response soon after damage/surgery. Additionally, drugs targeting Il-11 can be utilised and could hold promise as a potential therapeutic strategy for mitigating heterotopic bone formation.

In conclusion, our investigation unveils for the first time in zebrafish the occurrence of injury-induced heterotopic bone formation, akin to human MOT. The cumulative observations of heterotopic bone formation in the pectoral fin and caudal peduncle region and consistent increase in the size of the intermuscular bones following injury suggest that osteo-induction and growth of ectopic bone can occur in the zebrafish if appropriate injury methods are employed. Furthermore, with the ability to induce consistent changes in bone structure after injury and the subsequent imaging options available, the zebrafish holds without a doubt the potential to serve as a powerful and dependable experimental model for further research into heterotopic ossification. Using this model, we observed a significantly increased magnitude of heterotopic bone formation in zebrafish with a gain-of-function mutation in the Kcnk5b channel. This finding suggests a central role for potassium channel signalling, particularly through Kcnk5b, in regulating the skeletogenic injury response. In contrast, zebrafish carrying a loss-of-function mutation of the interleukin 11 receptor paralogue (Il11ra), known to be associated with impaired regeneration, exhibited a substantially reduced response. This contrast underscores the importance of interleukin signalling via Il11ra in the injury response mechanism leading to heterotopic bone formation. These findings not only advance our understanding of the molecular basis of heterotopic bone formation, but also provide potential insights into therapeutic strategies for human patients grappling with this debilitating condition.

## MATERIALS AND METHODS

### Zebrafish husbandry

Zebrafish work was conducted at the fish facilities of Lee Kong Chian School of Medicine (LKCMedicine), Nanyang Technological University (NTU), Singapore, and Boston Children's Hospital (BCH), USA. All experimental procedures involving fish adhered to Association for Assessment and Accreditation of Laboratory Animal Care (AAALAC) standards and were granted approval by the Institutional Animal Care and Use Committees (IACUC) of NTU under the protocol number A19028, and by the BCH IACUC under the protocol number 00001704. AB wild-type fish at the LKCMedicine fish facility were used to establish the injury model and perform RNA sequencing for transcriptomic analysis, and subsequent validation of the results. Furthermore, *Tg(sp7:egfp; ctsk:dsred)*, reporting osteoblasts in green and osteoclasts in red, and *casper* mutants were used for live imaging. *kcnk5b^dt30mh/+^* mutants, *il11ra^bns251^* mutants and their wild-type sibling controls housed at the BCH fish facility were used for later experiments. All fish were maintained in facility water at 28°C, adhering to a 14-h light and 10-h dark cycle.

### Injury experiments

The contusion setup comprised a 45° wedge and a customised lancing device (OneTouch, LifeScan, USA) with the depth setting typically adjusted to less than 1 mm, all positioned on a Petri dish (Fig. S1). The sharp tip of the lancet was replaced with a custom epoxy resin spheroid to prevent piercing the skin, instead causing a blunt injury. Adult zebrafish aged over 3 months post fertilisation were anesthetised with 0.013% tricaine (buffered to pH 7.0). Following anaesthesia, the fish were placed laterally on the Petri dish, ensuring that the target area rested on the impactor tip, and then subjected to five to ten lancet strikes of similar force conducted under direct visualisation using a Leica MZ7.5 dissecting stereomicroscope to induce a visible contusion. Early resolution of the contusion was addressed by gently stroking the injury site with a blunt-tip K-pin twice at 48-h intervals after the first injury, reverting it to its post-first-injury state. This process allowed the contusion phase to be sustained for up to 1 week. Regarding thoracic contusions, the lancing device setup was not used. Instead, all three injuries were induced by gently stroking the region with the blunt-tip K-pin, resulting in similar contusions as observed in the caudal peduncle region.

For pectoral fin injuries, following anaesthesia, fish were positioned on a Petri dish in a lateral orientation with the right side facing up. Subsequently, the pectoral fin was raised to expose the medial muscle bulk, and gentle stroking using a Dumont #5 forceps was carried out at the mid-section of this muscle bulk, guided by tactile feedback from the forceps tip as it grazed over the surface of the bone. This process resulted in muscle injury and microscopic damage to the pectoral fin rays. Both procedures were performed under a Leica MZ7.5 dissecting stereomicroscope. No signs of distress were observed post injuries in any of the fish.

### Alizarin Red staining and imaging

Alizarin Red S (Sigma-Aldrich, A5533) staining of euthanised fish to visualise the skeleton was performed based on the protocol described by Schilling (2002). For live staining, the fish were housed overnight in small tanks containing 200 ml of fish water with 0.001% Alizarin Red (Tomecka et al., 2019). Before imaging, they were transferred to clean water for a brief rinse and then anaesthetised. Brightfield imaging was performed using an Axio Zoom V16 (Zeiss) stereo zoom microscope. Measurements of bones, when necessary, were conducted using Zen Blue 3.3 or Zen Lite 2.3 image analysis software by Carl Zeiss.

### RNA sequencing

Tissues were harvested from the caudal peduncle site before and after injury from different sets of fish. Injuries were done in a similar manner as described above. Each condition, i.e. no injury, 24 h after one injury, 24 h after three injuries and 5 days after three injuries, had three replicates. Each replicate was from three adult fish, using approximately 0.5 cm muscle tissue from the caudal peduncle region proximal to the tail fin, taken after removal of the skin. Harvested sample was predominantly muscle tissue except for the tiny intermuscular bones that could be present. RNA was extracted as per a previously published protocol (Peterson and Freeman, 2009). Initial assessment of RNA concentration and purity was conducted using a NanoDrop spectrophotometer (Thermo Scientific). The RNA integrity number was subsequently assessed with an Agilent 2100 Bioanalyzer, confirming that all samples met the necessary quality standards for sequencing. The mRNA library preparation and paired-end PE150 sequencing was carried out on the Illumina Novaseq-6000 platform by NovogeneAIT Genomics Singapore. Following quality control and pre-processing, the clean reads were mapped to the Zebrafish Genome Assembly GRCz11 (GCA_000002035.4) using *salmon* (v.1.9.0) (Patro et al., 2017), considering decoys and GC biases for zebrafish with an average of 83.8% (range: 82.1-86.1%) fragment hits. All downstream data analyses were carried out in R/RStudio. Gene expression levels under different conditions were noted and the correlation of gene expression levels among the 12 samples was assessed using Pearson's correlation coefficient. Although replicates had high similarity (R^2^>0.97) showing sample reproducibility, differential expression was noted among the four conditions (R^2^ range: 0.74-0.91). The *DESeq2* package (v.1.38.3) was used for differential analysis of count data, where lowly expressed counts (<10) were filtered out from the dataset (Love et al., 2014). The *clusterProfiler* (v.4.6.0) package was used for GO and KEGG pathway analysis (Wu et al., 2021). All figures were generated using *ggplot2* (Valero-Mora, 2010).

### qRT-PCR

For qRT-PCR validation, cDNA conversion was done using 1 µg of DNase-treated RNA sample, oligo-dT (Thermo Fisher Scientific), MultiV Reverse Transcriptase (RT) enzyme (New England Biolabs), 10× RT buffer (New England Biolabs) and 10 mM dNTP mix (New England Biolabs). All qPCR reactions were carried out on an Applied Biosystems Step One Plus real-time PCR system using standard thermal-cycling conditions optimised for KAPA SYBR FAST qPCR Mix (Kapa Biosystems). The primer sequences used were as follows: *kcnk5b* forward, 5ʹ-ATCACTCTCCTCGTCTGCAACG-3ʹ, and reverse, 5ʹ-GAGTCCCATGCACAACGTGCAG-3ʹ; *il11a* forward, 5ʹ-GGACAAATATGAAATTGCTGGGTG-3ʹ, and reverse, 5ʹ-AGCGTCAGAAGGAGTTTGGT-3ʹ; *il11b* forward, 5ʹ-TGAACGCAAATGAGTTGACTG-3ʹ, and reverse, 5ʹ-CCCAATTCGTCACTATTCCGT-3ʹ; *rpl13a* forward, 5ʹ-AGACGCACAATCTTGAGAGCAG-3ʹ, and reverse, 5ʹ-TCTGGAGGACTGTAAGAGGTATGC-3ʹ. Data were exported as Excel files and analysed using the 2^−ΔΔCT^ method (Livak and Schmittgen, 2001).

### Micro-CT scans

Micro-CT scans were performed on injured and uninjured pectoral fins of *kcnk5b and il11ra* mutants, as well as their wild-type controls. After routine Alizarin Red staining and two-dimensional imaging, the fins were stained with 1% silver nitrate in multi-well cell culture dishes placed on a standard gel lightbox for a duration ranging from 45 min to 1 h. The actual duration was determined based on the degree of staining observed in fin rays and the background intensity. After adequate staining, the samples were washed with distilled water three times and then fixed onto agar blocks and stored at 4°C until scanning. Scanning was done using the Skyscan 1173 micro-CT scanner (Bruker). Following the scan, volume-rendered images of the contrast-stained samples were created using the Amira software package, version 6.0 (Thermo Fisher Scientific), and saved as NIfTI files. Subsequently, volumetric assessments were done using 3D slicer (Kikinis et al., 2014).

### Statistical analysis

All statistical analyses were performed using R and GraphPad Prism 9 (GraphPad Software, San Diego, CA, USA) as deemed appropriate. Before comparing continuous variables, the assumption of normal distribution was assessed using the Shapiro–Wilk test. In cases where the data exhibited a normal distribution, Student's *t*-test was used for comparison. Conversely, when the data did not follow a normal distribution, the Mann–Whitney *U-*test was used to ascertain statistical significance. For evaluating associations between categorical variables, Fisher's exact test was employed. A *P*-value of less than 0.05 was considered statistically significant, unless otherwise stated.

## Supplementary Material

10.1242/dmm.050724_sup1 Supplementary information
