# Supplementary material for: Genetic regulation of injury-induced heterotopic ossification in adult zebrafish
Source: Dis Model Mech. 2024 May 31;17(5):dmm050724. doi: 10.1242/dmm.050724 (PMC11152560; doi:10.1242/dmm.050724)
Supplement: Supplementary information [file dmm-17-050724-s1.pdf]

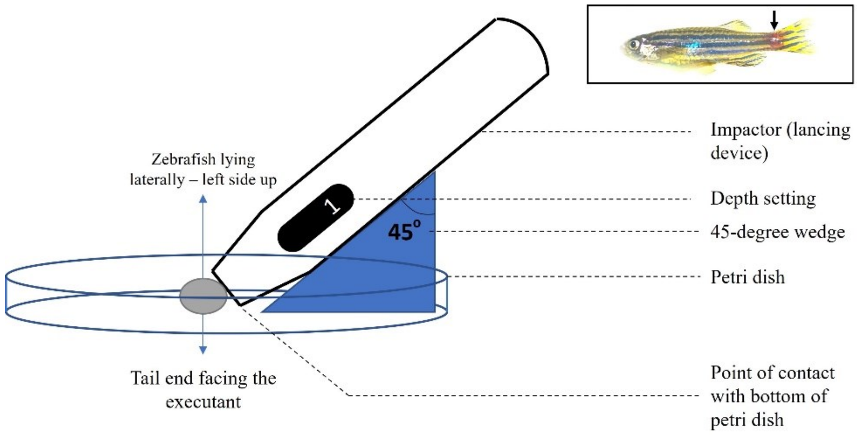

Fig. S1. Setup for creating caudal peduncle contusion using the lancing device (OneTouch, LifeScan Inc., USA), which incorporates a lancet with its sharp tip replaced by a custom epoxy resin spheroid, alongside a 45-degree wedge placed on a petri dish. The boxed area highlights an adult zebrafish post-contusion (arrow).

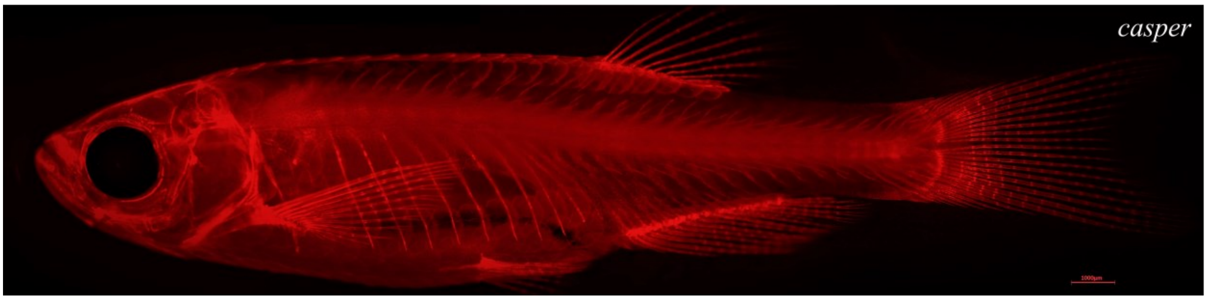

Fig. S2. Alizarin red stained *casper* mutant zebrafish showing the entire ossified skeleton in bright red fluorescence. Scale bar:1000µm.

Disease Models & Mechanisms • Supplementary information

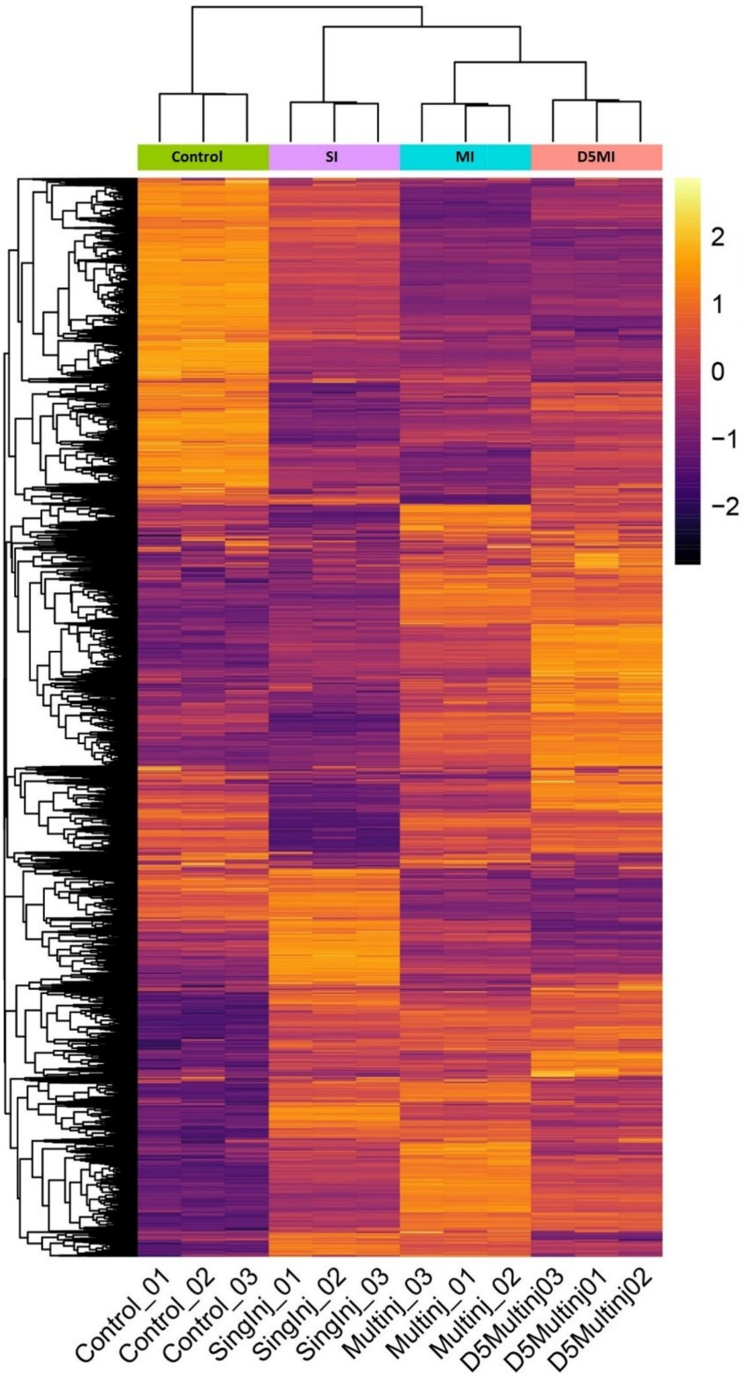

Fig. S3. Heat map of the top 10,000 differentially expressed genes (DEGs), revealing a temporal relationship with expression profiles of the D5MI group more closely related to the MI group when compared to the SI group and Control group, which were the most distantly related.

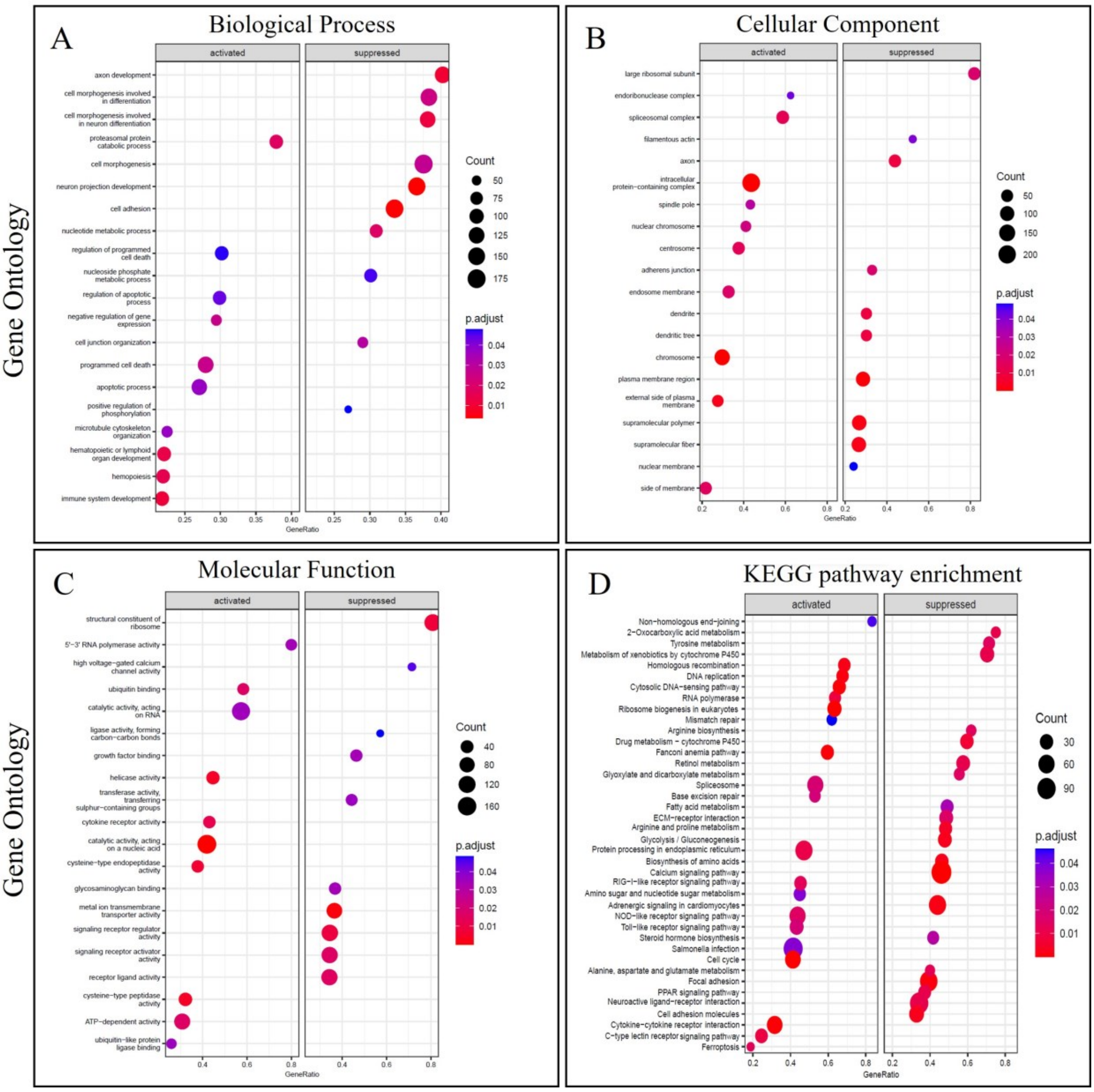

Fig. S4. Functional annotations based on GO terminology and KEGG pathway enrichment for comparison 1 (Single injury vs. Control). **A)** Biological process. **B)** Cellular component. **C)** Molecular function and **D)** KEGG Pathway Enrichment.

Disease Models & Mechanisms • Supplementary information

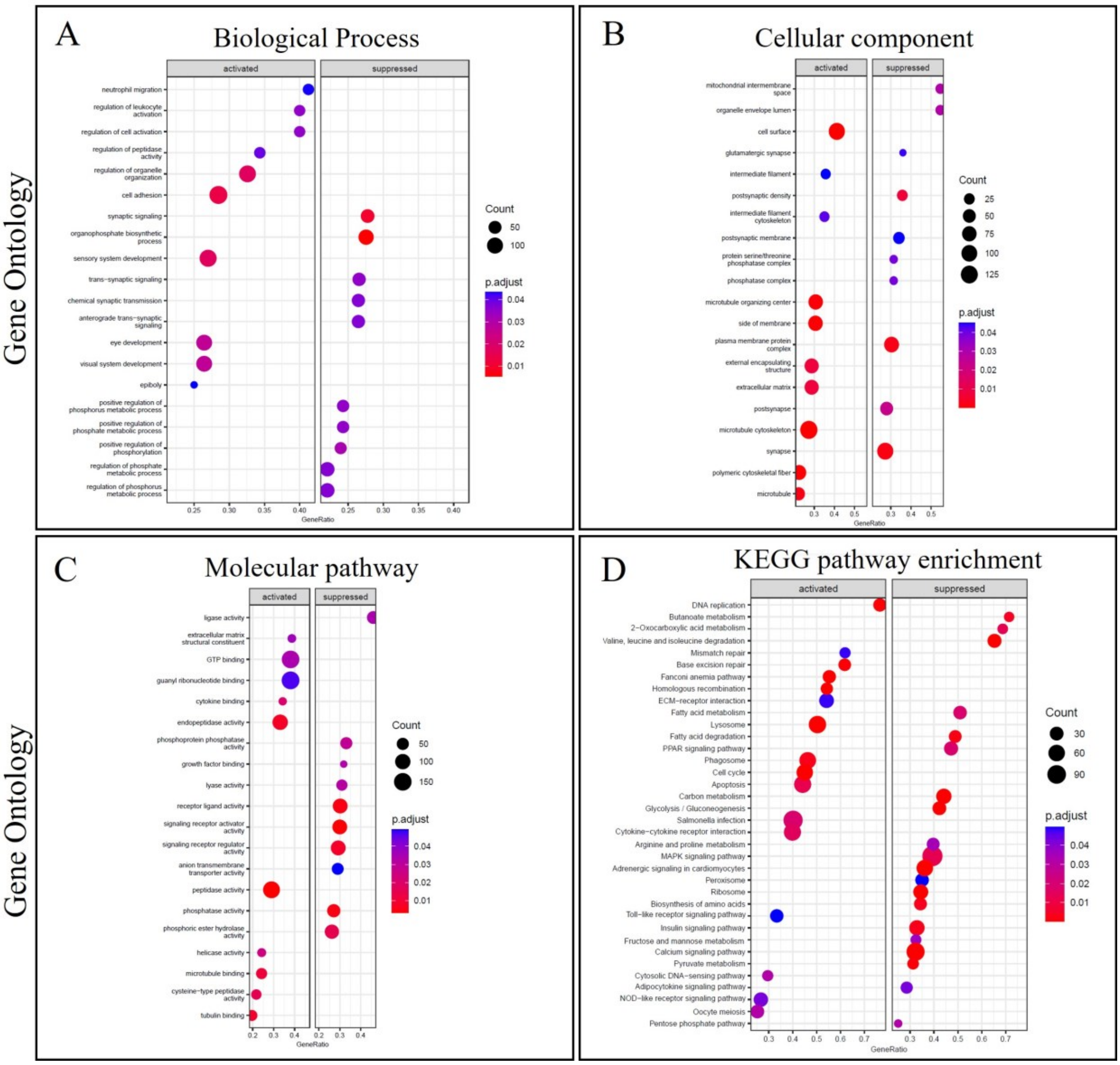

Fig. S5. Functional annotations based on GO terminology and KEGG pathway enrichment for comparison 2 (Multiple injuries vs. Control). A) Biological process. B) Cellular component. C) Molecular function and D) KEGG Pathway Enrichment.

Disease Models & Mechanisms • Supplementary information

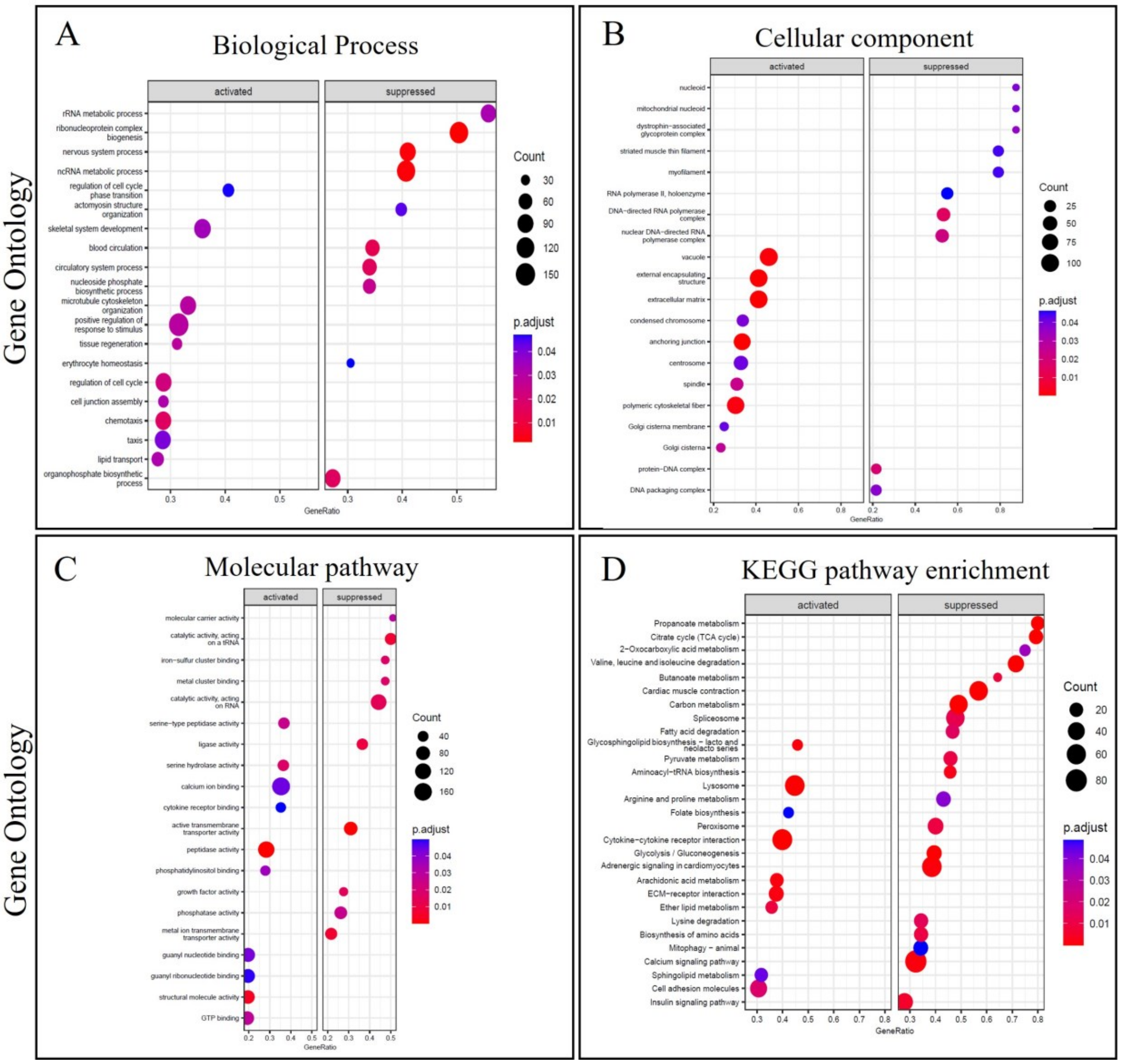

Fig. S6. Functional annotations based on GO terminology and KEGG pathway enrichment for comparison 3 (5 Days after multiple injuries vs. Control) A) Biological process. B) Cellular component. C)Molecular function and D) KEGG Pathway Enrichment.

Table S1. Statistically significant differentially expressed genes for comparison 1 (Single injury vs. Control).

Available for download at
https://journals.biologists.com/dmm/article-lookup/doi/10.1242/dmm.050724#supplementary-data

Table S2. Statistically significant differentially expressed genes for comparison 2 (Multiple injuries vs. Control).

Available for download at
https://journals.biologists.com/dmm/article-lookup/doi/10.1242/dmm.050724#supplementary-data

Table S3. Statistically significant differentially expressed genes for comparison 3 (5 Days after multiple injuries vs. Control).

Available for download at
https://journals.biologists.com/dmm/article-lookup/doi/10.1242/dmm.050724#supplementary-data

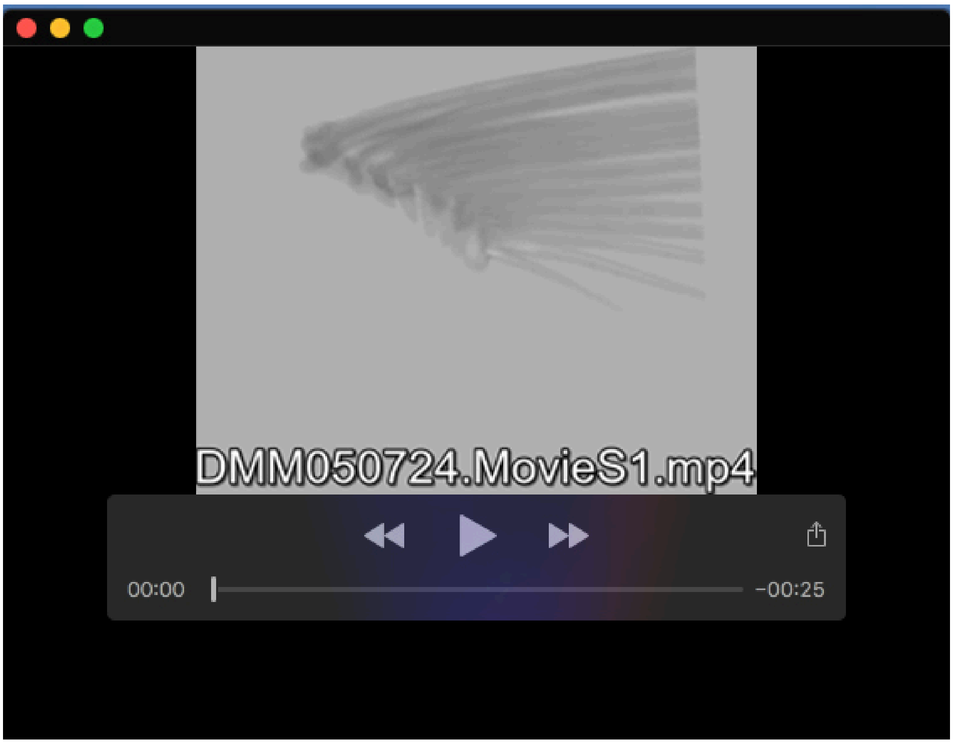

Movie 1. 3D reconstructed CT scan of an uninjured left pectoral fin in *kcnk5b*$^{pfau/+}$ mutant zebrafish.

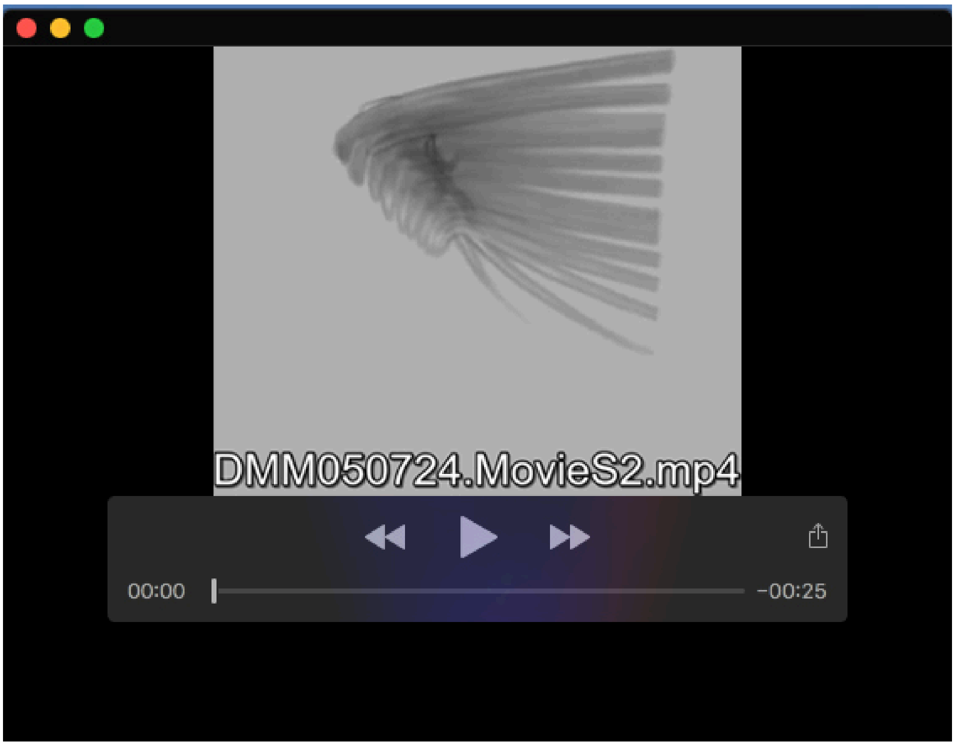

Movie 2. 3D reconstructed CT scan of an injured right pectoral fin in *kcnk5b*$^{pfau/+}$ mutant zebrafish, revealing extensive heterotopic ossification on the medial aspect.

Disease Models & Mechanisms • Supplementary information

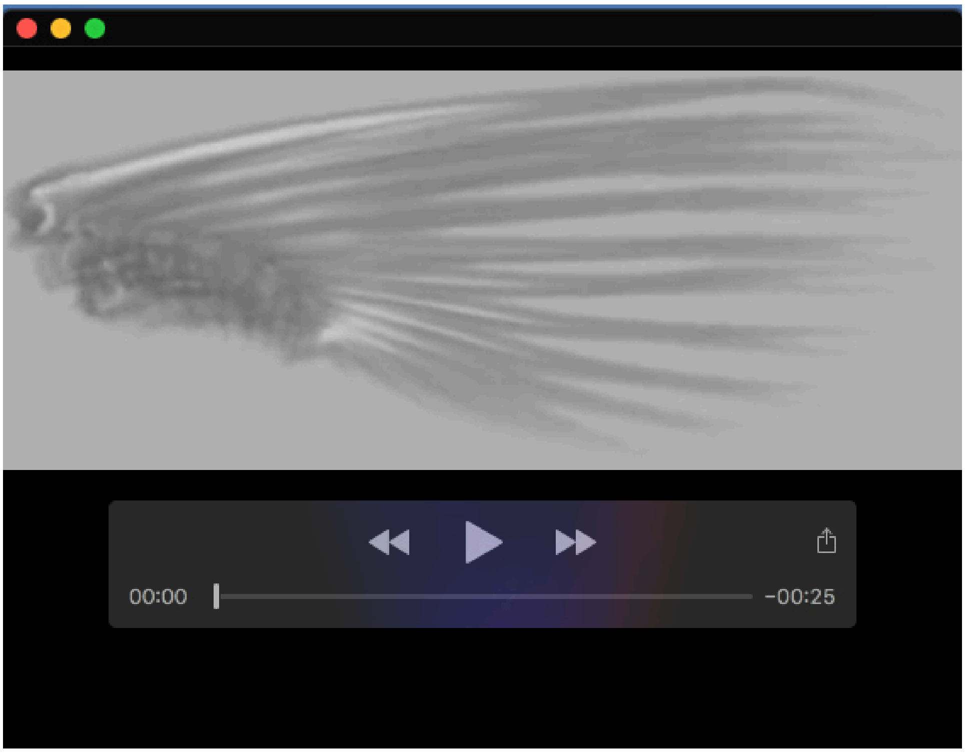

Movie 3. 3D reconstructed CT scan of an uninjured left pectoral fin in *il11ra*$^{-/-}$ mutant zebrafish.

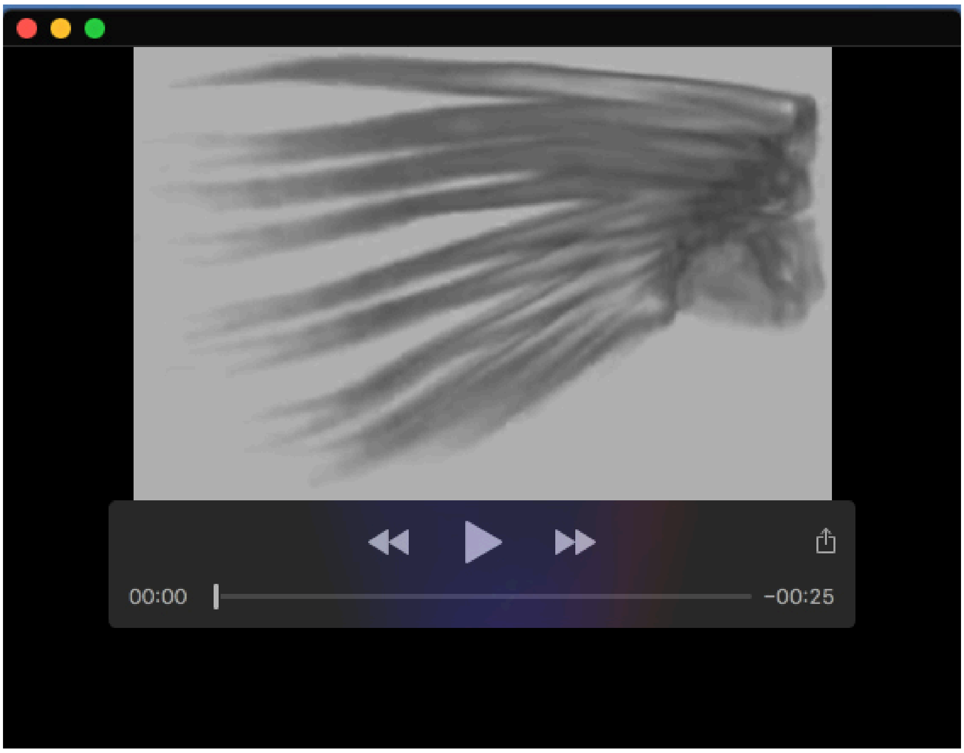

Movie 4. 3D reconstructed CT scan of an injured right pectoral fin in *il11ra*$^{-/-}$ mutant zebrafish, showing no signs of heterotopic bone.
